# Transcranial direct current stimulation (tDCS) to dorsolateral prefrontal cortex influences perceived pleasantness of food

**DOI:** 10.1016/j.heliyon.2023.e13275

**Published:** 2023-02-04

**Authors:** Eric C. Anderson, Julie A. Cantelon, Amanda Holmes, Grace E. Giles, Tad T. Brunyé, Robin Kanarek

**Affiliations:** a Center for Applied Brain and Cognitive Sciences, Medford, MA, 02155, USA; b Center for Interdisciplinary Population and Health Research, MaineHealth Institute for Research, Portland, ME, 04101, USA; c Tufts University School of Medicine, Boston, MA, 02111, USA; d Tufts University, Medford, MA, 02155, USA; e Cognitive Science and Applications Team, DEVCOM Soldier Center, Natick, MA, 01760, USA

**Keywords:** Brain stimulation, tDCS, Craving, Liking, Wanting

## Abstract

The ability to regulate the intake of unhealthy foods is critical in modern, calorie dense food environments. Frontal areas of the brain, such as the dorsolateral prefrontal cortex (DLPFC), are thought to play a central role in cognitive control and emotional regulation. Therefore, increasing activity in the DLPFC may enhance these functions which could improve the ability to reappraise and resist consuming highly palatable but unhealthy foods. One technique for modifying brain activity is transcranial direct current stimulation (tDCS), a non-invasive technique for modulating neuronal excitability that can influence performance on a range of cognitive tasks. We tested whether anodal tDCS targeting the right DLPFC would influence how people perceived highly palatable foods. In the present study, 98 participants were randomly assigned to receive a single session of active tDCS (2.0 mA) or sham stimulation. While receiving active or sham stimulation, participants viewed images of highly palatable foods and reported how pleasant it would be to eat each food (liking) and how strong their urge was to eat each food (wanting). We found that participants who received active versus sham tDCS stimulation perceived food as less pleasant, but there was no difference in how strong their urge was to eat the foods. Our findings suggest that modulating excitability in the DLPFC influences “liking” but not “wanting” of highly palatable foods. Non-invasive brain stimulation might be a useful technique for influencing the hedonic experience of eating but more work is needed to understand when and how it influences food cravings.

## 1 Introduction

Food cravings are a strong desire for specific foods, often ones that are calorie dense. Food cravings are extremely common (experienced by 21–97% of the adult population) [1], and have important health implications. Cravings are linked to greater body mass index [2], predict weight regain after bariatric surgery in patients who are obese [3], and are associated with early attrition from weight loss programs [4]. As rates of global obesity rise [5], it is important to understand why some individuals can exert self-control and maintain healthy dietary choices, while others cannot inhibit hedonic, reward-driven eating.

Previous neuroimaging work has demonstrated the importance of the prefrontal cortex, specifically the dorsolateral prefrontal cortex (DLPFC), in effortful regulation of eating behavior and hedonic response to food stimuli [[6], [7], [8]]. The DLPFC is involved in cognitive control processes, such as reappraisal and inhibition, that help people to resist consuming highly palatable but unhealthy foods [9,10].

Previous work has utilized noninvasive brain stimulation to modulate DLPFC activity and test whether cravings can be modified [11]. Transcranial direct current stimulation (tDCS) and repetitive transcranial magnetic stimulation (rTMS) are two common non-invasive brain stimulation techniques. While each of these modalities has distinct strengths, the simplicity and affordability of tDCS makes it appealing for scaling-up clinical interventions to modify cravings. Most tDCS applications use a bipolar electrode montage including a single anodal and single cathodal electrode to administer low-intensity (e.g., 0.5–2.0 mA) direct current to underlying cortical tissue [12]. According to sliding-scale models of tDCS, stimulation causes cortical regions underlying the anodal electrode to be upregulated (neuronal membrane depolarization) whereas regions underlying the cathodal electrode are downregulated (neuronal membrane hyperpolarization [13].

Research targeting the right DLPFC with 2.0 mA anodal current has shown significant reductions in subjective cravings in both normal and overweight individuals, in comparison to sham control conditions [[14], [15], [16], [17], [18]]. However, inconsistencies have also been reported, with some studies revealing no anti-craving effects when right anodal DLPFC stimulation is combined with food-related inhibitory control training [19], or when a left anodal stimulation is used [14]. One possibility is that differences in individual participants’ psychological and physiological states and traits might contribute to the observed variability. While several meta-analyses have found the majority of evidence supports the influence of inhibitory neuromodulation (rTMS and tDCS) of the DLPFC in diminishing cravings in general [16,20,21], the evidence that tDCS in particular can reduce food cravings is much less clear [22]. Therefore, further well-powered research is warranted.

Additionally, prior research has not addressed several important factors. First, previous research using tDCS has focused broadly on cravings for food. However, craving responses have been conceptualized as two related constructs that guide eating behavior: ‘liking’ and ‘wanting’ [[23], [24], [25]]. Liking is the pleasant hedonic/affective experience of consumption, while wanting is the incentive/motivational drive to consume a food reward. These constructs often co-occur, but different brain regions are activated by the hedonic state of liking (orbitofrontal cortex) verses the motivational state of wanting (amygdala) [26,27]. Experimental procedures have been developed to dissociate the two constructs in laboratory settings [28]. For instance, asking participants to rate “how pleasant would it be to eat this food?” and “how strong is your urge to eat this food” may allow for assessment of the relative contributions of each of these constructs. Distinctly measuring how tDCS can influence these two processes has not been done before, to our knowledge, and can provide better insights into the how tDCS can be used to modify food craving and consumption.

Additionally, previous research has not extensively examined how individual differences (states and traits) may modulate the effects of tDCS. For instance, traits related to self-control, craving regulation, and restrained dietary intake might also moderate the effects of tDCS. Specifically, individuals who endorse restricting food intake are more sensitive and reactive to food cues than unrestrained eaters, demonstrating significantly greater craving, liking and desire to eat a cued food following pre-exposure [29]. Furthermore, research has demonstrated that individuals who displayed more reflective choice behavior are more susceptive to the anti-craving effects of tDCS than those who displayed more impulsive choice behavior [17]. Thus, restrained eaters, who display more reflective choice behavior, may show greater effects of tDCS on food cravings than unrestrained eaters.

In the present study, we extend previous findings by evaluating whether anodal tDCS stimulation of the DLPFC specifically influences liking and/or wanting of highly palatable foods. We hypothesized that participants receiving active stimulation would have significant reductions in subjective ratings of liking and wanting of highly palatable food images in comparison to participants in the sham condition. We also explored whether the individual differences subjective hunger, self-reported dietary restraint, and state/trait cravings would modulate the effect of tDCS stimulation.

## 2 Methods

To encourage transparency and reproducibility, we report how we determined our sample size, all data exclusions (if any), all manipulations, and all measures in the study (see online supplemental materials for complete list of measures collected) [30]. Lead author has data reported in the manuscript and R code for analyzing the data which is available upon request.

### 2.1 Participants

An a priori sample size was determined based on previous findings and practical limitations. We estimated the effect size of active versus sham tDCS based Jansen et al.'s meta-analysis which found brain stimulation (compared to control) reduced cravings for food, alcohol, and drugs with an effect size of approximately 0.5 (Hedge's g = 0.48) [16]. Since previous work motivated a directional hypothesis (cravings during active brain stimulation would be reduced compared to sham stimulation), we used one-tailed confirmatory tests. Using this effect size and achieving 75% power would require 98 participants for a one-tailed test (G*Power version 3.1.9.2). While greater power would be ideal, our sample size was also constrained by practical considerations (study budget and personnel time). It is worth noting that n = 98 is quite large for tDCS studies of this type (largest previous n = 31) [22]. This study was approved by the Institutional Review Board at Tufts University, with secondary approvals by the U.S. Army (both of whom financially supported this work). All participants were informed about the study, and gave written informed consent before participating. Participants received $20 for remuneration.

As in previous studies [31], we recruited female participants with frequent cravings (more than 3 times per week). Potential participants were also screened to meet tDCS eligibility requirements: no history of adverse responses to tDCS; no participation in tDCS study in the last year; no history of seizures, head injuries, neurological/psychiatric disorder; no metal in the head; and no scalp sensitivity. We recruited 102 female participants. Four did not complete the study due to discomfort during tDCS (see below), leaving a final sample of 98 (see Table 1 for participant characteristics).

**Table 1:** Participant information.

Characteristic	Active, N = 49*1*	Sham, N = 49*1*	p-value*2*
Age	20.4 (2.7)	20.8 (3.5)	0.9
Height (inches)	65.6 (3.0)	65.8 (3.3)	0.8
Weight (lbs.)	138.3 (21.8)	138.8 (19.4)	0.8
Body Mass Index	22.6 (3.3)	22.6 (3.7)	>0.9
Subjective hunger	28.0 (20.5)	37.5 (23.0)	0.043
Hours since meal	6.0 (6.2)	6.8 (5.9)	0.2
Control of eating behavior	10.6 (4.5)	8.4 (4.7)	0.021
Disinhibition of control	8.4 (2.8)	7.6 (2.9)	0.2
Susceptibility to hunger	6.6 (3.1)	6.6 (2.7)	>0.9
Trait cravings	68.9 (16.0)	67.7 (16.4)	0.5
State cravings	36.1 (11.5)	37.5 (13.1)	0.6
Time of day			0.7
Morning	30/49 (61%)	28/49 (57%)	
Afternoon	19/49 (39%)	21/49 (43%)	
Guessed condition			0.066
Active	22/48 (46%)	13/47 (28%)	
Sham	26/48 (54%)	34/47 (72%)	

Statistics presented: mean (SD); n/N (%).

Statistical tests performed: Wilcoxon rank-sum test; chi-square test of independence.

Participants were not required to answer questions, so N is smaller than complete sample.

### 2.2 Study overview

This section provides a brief overview of the entire study session. Further details are provided in the sections below. To maximize the evocativeness of the food stimuli, experimental sessions were scheduled to start during times when participants were more likely to be hungry: in the morning between 10am and 12pm and during the afternoon from 3pm to 5pm (Table 1). After participants consented to take part in the study, research staff verified their tDCS eligibility criteria. Eligible and consenting participants then completed the study questionnaires on a tablet. While participants completed the questionnaires, the tDCS electrodes were placed on their heads in accordance with the international 10/20 system coordinates (F4 for anodal electrode, F3 for cathodal electrode), but stimulation was not started. Participants then completed the “baseline” phase of the picture task, which involved rating pictures (detailed below). The tDCS device was then turned on, and participants received active or sham stimulation for the following 20 min. Active stimulation ramped up current intensity to the target (2.0 mA) over the course of 30 s, and then maintained that intensity for the duration of stimulation; the device ramped down current intensity over the final 30 s of stimulation. Sham stimulation followed the common ramp-up/ramp-down procedure involving 30 s of ramping-up to the target intensity (2.0 mA), then 30 s of ramping-down and stopping stimulation for the duration of the session. Participants then completed an eye tracking task (data not reported in this manuscript). Next participants completed the tDCS “stimulation” phase picture task (identical to the previous version). Finally, stimulation was stopped, the tDCS equipment was disconnected, participants were remunerated and debriefed.

### 2.3 Picture task

Each participant completed the same picture task twice: during the “baseline” phase before tDCS was applied, and during the “stimulation” phase (with active or sham tDCS). During each picture task, participants viewed the same 40 food pictures from the ‘Food-pict’ database presented in a random order [32]. The pictures used in this study were rated as frequently craved by previous participants [32]. These pictures included fruits, desserts, and snacks (a complete list of stimuli used are included in Supplemental Online Materials). In the present study, for each picture, participants made two ratings on 100-point scales. To measure liking, the first question asked: “How pleasant would it be to eat this”? (1 = Very unpleasant; 100 = Very pleasant). To measure wanting, the second question asked “How strong is your urge to eat this”? (1 = No urge to eat; 100 = Extremely strong urge to eat). Pictures remained on the screen until participants made both ratings and advanced to the next picture.

### 2.4 tDCS stimulation

To maintain consistency with previous studies, we targeted the right dorsolateral prefrontal cortex, using a common montage: anode F4 (right), cathode F3 (left) [15]. tDCS was administered using the M × N high-definition tDCS system manufactured by Soterix Medical, Inc. (New York, NY). Two Ag/AgCl sintered ring electrodes (30 cm diameter) and plastic electrode holders were inserted into a 74-channel EasyCap (EEG cap) sized and fitted to each participant. The electrode holders positioned the electrodes approximately 7 mm above the surface of the scalp, and Signa gel (Parker Laboratories, Fairfield, NJ) was used to conduct current between the electrode and scalp to reduce impedance. During setup, we ensured that impedences were lower than 2 kΩ, achieving which often involved additional scalp cleaning (with alcohol), hair parting, and conductive gel.

Participants were randomly assigned to receive either “active” or “sham” stimulation based on the order in which they participated. Experimenters were not blinded to condition due to limited staffing (one research assistant read the assigned condition, set up equipment, and administer the study). In the active stimulation condition, current was linearly ramped up over 30 s from 0 mA to the full stimulation of 2 mA. Stimulation stayed at this level for 20 min. In the “sham” stimulation condition, the current was linearly ramped up for 30 s from 0 mA to 2 mA, and then linearly ramped down for 30 s to 0 mA and remained that way for the remainder of the study. To evaluate perceived cutaneous sensation differences across active and sham tDCS groups, after 30 s, participants were asked to indicate how the tDCS felt on a 10-point scale (0 = Cold to 9 = Hurts a lot) [33]. Of the original 102 participants, 4 reported some discomfort (≥7 on the scale), so stimulation was stopped as specified by our protocol, and these participants were removed from analysis. After at least 5 min of tDCS, participants completed the “stimulation” phase picture rating task. At the end of the study, participants were asked to guess which condition they were in (active or sham).

### 2.5 State and trait measures

To explore how individual differences in state and trait factors might modulate the effects of tDCS, participants were asked to complete a set of questionnaires before tDCS stimulation. Participants answered a single-item subjective hunger question on a 100-point scale: “How hungry do you currently feel”? (0 = not at all hungry; 100 = very hungry). Study subjects were asked to complete the 36-item Food Craving Questionnaire [34], which yields a trait cravings factor and a state cravings factors, as well as a 52-item Three Factor Eating Questionnaire [35] which yields three factors of restrained eating: Factor I = ‘cognitive control of eating behavior’; Factor II = ‘disinhibition of control’; Factor III = ‘susceptibility to hunger’. Due to an error in creating the questionnaires, two response items (questions 48 & 49) were removed from the Three Factor Eating Questionnaire. This results in scores that would be slightly lower than what has been observed in other studies. However, because we are interested in variation between participants, the current results will allow comparisons between participants within this study. Participants were given the option of not answering any question, so the number of participants who completed different questions varies (Table 1).

### 2.6 Statistical analysis

To test hypotheses about the effects of tDCS, we computed change scores in participants’ ratings during the picture task (tDCS stimulation phase minus baseline phase) and then computed means across the 40 picture stimuli presented in each phase. We examined the changes in ratings using planned independent samples t-tests (active vs sham). To explore how individual state and trait differences might be related to the effect of tDCS on liking and wanting of food, we explored the relationship between hunger, restrained eating, cravings, and the effect of tDCS. These analyses were exploratory but theoretically motivated. For instance, but we reasoned that hungry participants might be more likely to be influenced by tDCS because they have stronger cravings for food. Linear models were used with changes in ratings as the dependent variable. Different models were conducted for each state/traits (hunger, restrained eating, cravings), all of with included the tDCS condition (active vs. sham), and the interactive effects between these variables (e.g. hunger * condition) were entered as the predictors. R version 3.6.2 was used for all analysis.

## 3 Results

**Fig. 1:**
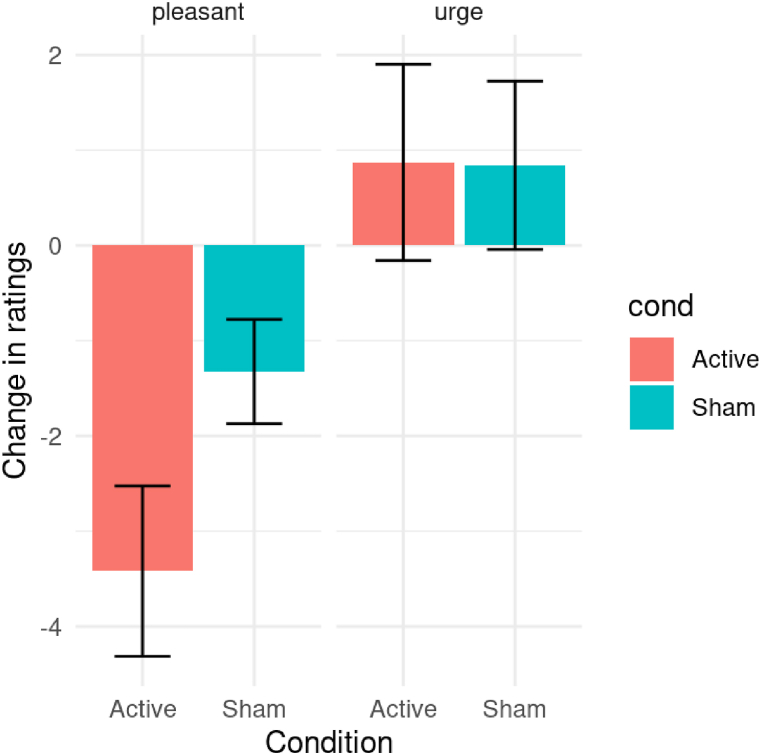
The effect of tDCS on reported pleasantness and urge to eat Notes: Error bars represent standard errors. Difference scores calculated as ratings during the stimulation phase (active or sham) minus ratings during baseline phase (original scale 0 = not very, 100 = very).

We found that participants receiving active tDCS stimulation had significantly greater reductions in pleasantness ratings (M = −3.42, SD = 6.26) than participants receiving sham stimulation (M = −1.33, SD = 3.84), t(79.62)=−2.00, p=.025, *d* = −0.40, 95% CI [-0.81, 0.002]. For urge to eat ratings, we found no significant difference between participants receiving active tDCS stimulation (M = 0.87, SD = 7.21) and participants receiving sham stimulation (M = 0.84, SD = 6.19), t(93.84)=0.02, p=.509, *d* = 0.004, 95% CI [-0.40, 0.41]. In short, at baseline there was little difference between the groups ratings of pleasantness or urge to eat (Fig. 1; raw ratings are presented in the online supplemental materials). But after receiving tDCS to DLPFC, the group receiving active stimulation reported food would be less pleasant to eat. tDCS did not change participants’ urge to eat.

Next we conducted exploratory analyses between individual state and trait differences and the effect of tDCS on liking and wanting of food. At baseline, correlational analyses showed a strong relationship between self-reported hunger and both pleasantness (r=.32, 95% CI [.11, .50], t(82)=3.01, p=.003) and urge to eat (r=.48, 95% CI [.30, .63], t(82)=4.94, p<.001), before any tDCS stimulation. Hungry participants had higher ratings of pleasantness and urge to eat than less hungry participants (see supplemental materials for scatter plot of relationships).

Next, we explored the relationship between hunger and the effect of tDCS on food ratings. In particular, we were interested in whether level of hunger modulated the effect of tDCS. In this analysis, neither the main effects nor interaction were significant for ratings of pleasantness (all ps > .24) or urge to eat (all ps > .34)

Next, we explored the relationship between the three factors of restrained eating [35] and the effect of tDCS on food ratings. As above, we were interested in whether any of the three factors modulated the effect of tDCS. We ran one model for each factor of restrained eating (cognitive control over eating; controlling urges; susceptibility to hunger). In this analysis neither the main effects nor interactions were significant for ratings of pleasantness (all ps > .17) or urge to eat (all ps > .26) for any of the three factors.

Finally, we explored the relationship between the state and trait craving [34] and the effect of tDCS on food ratings. As above, we were interested in whether either factor modulated the effect of tDCS. We ran one model for each craving factor (i.e., state and trait). In this analysis neither the main effects nor interactions were significant for ratings of pleasantness (all ps > .17) or urge to eat (all ps > .12) for either of the two craving factors. In short, the effect of tDCS was not modulated by any of the traits or states measured (see supplemental material for more details).

## 4 Discussion

The ability to regulate intake of unhealthy foods is critical in modern, calorie dense food environments. The present study examined the effects of a single session of tDCS to the DLPFC to reduce cravings of palatable foods. Consistent with the first part of our hypothesis, participants receiving active tDCS stimulation had reduced ratings of liking compared to participants receiving sham stimulation. However, in contrast to the second part of our hypothesis, active tDCS stimulation did not change ratings of wanting compared sham stimulation. It is not clear why one construct was influenced and the other was not.

Separately measuring liking and wanting was one of the unique contributions of the present study as previous studies have not distinguished between these constructs. For instance, most previous tDCS studies examining this topic have used a single visual analogue scale or the Food Craving Questionnaire [20]. However, studies do not always report the exact wording of questions, so it is difficult to assess exactly what is measured. Standardizing measures is an important future direction for research studying the effect of tDCS on cravings. The finding that tDCS influences liking but not wanting is somewhat counterintuitive since the construct of cravings measured in other work seems to most closely align with the construct of wanting. Thus, one might have predicted that wanting would be most influenced by tDCS while liking remained relatively unchanged. The present study found the opposite pattern of results.

This study also explored how participants' individual differences in states and traits might modulate the effect of tDCS. We found no evidence that the effects of tDCS was modulated by any of the six variables we tested (i.e., state subjective hunger; cognitive control of eating behavior; disinhibition of control; susceptibility to hunger; state and trait cravings). One possibility is that, in general, individual differences do not modulate the effects of tDCS; that is, effects are consistent across individuals regardless of the individuals’ transient states or more enduring traits. However, we believe this is unlikely given research showing that certain traits, such as spatial sense of direction, working memory capacity, and creativity can modulate tDCS effects on task performance [[36], [37], [38]]. Another possibility is that while the constructs we measured do not modulate the effects of tDCS, other constructs might. For instance, the constructs of cognitive control or ability to regulate thoughts and cravings might modulate the effect of tDCS. An additional possibility is that the modulate effects of the variables we measured might have been too small to detect with our sample size (though our sample size was quite large compared to other tDCS studies [22].

Understanding which individuals are influenced by tDCS remains an important direction for future work because using tDCS in applied settings will be more effective if individuals could be identified who would likely benefit—consistent with the idea of “precision medicine.” Future work should continue to interrogate why tDCS studies yield such variable findings. A deeper understanding the neurological and psychological mechanisms behind tDCS would be helpful in developing protocols to test how tDCS influences eating behavior in the lab and real world settings. For instances, differences in anatomical features (skull thickness, cerebral volume, etc.) might modulate the effect of tDCS and require different stimulation intensity and/or electrode placement. Finally, it will be important to move this work beyond small laboratory studies to test whether tDCS influences real world eating in a lasting way that has an impact on individuals’ health.

This study has a number of strengths and limitations. First, the study sample size was at least three times the size of previous tDCS studies that have explored this topic (largest previous n = 31) [22], and our study was based on an a priori power calculation. This study employed a between-subjects design in order to reduce demand characteristics associated with participants' ability to discern between experimental conditions [[39], [40], [41]]. This decision was based on previous tDCS research demonstrating that it is difficult to mask active versus sham tDCS conditions when utilizing repeated-measures designs due to cutaneous sensation differences at electrode sites [42]. However, the present study also has limitations. First, while this study had a larger sample size than many, we still only had 75% power to detect an effect size of Hedge's g = 0.476 using a one-tailed test comparing whether active tDCS resulted in lower cravings compared to sham stimulation. We had much less power to examine our exploratory goals related how individual state and trait differences might be related to the effect of tDCS on craving. While our between participant design was used to reduce participants' ability to discern which experimental condition they were in (active or sham tDCS), it does not appear that our blinding was completely successful. In particular, participants in the sham condition correctly guessed their condition 3 out of 4 times. Future work might reduce the potential influence of expectation-based effects by introducing a low-intensity sham condition that equates cutaneous sensation at the beginning and end with the active condition [42], or by increasing electrode size to reduce cutaneous sensation in the active condition. For practical reasons, research personnel were not blinded to the conditions, but this could have also created unintended differences in how they interacted with participants. Future work should use a double blinded approach. Additionally, our study included a relatively homogeneous sample of women, mostly college students, like most other studies on this topic. This may limit the ability to generalize these findings more broadly to other populations. Our study took place in the mornings (10–12 a.m.) and afternoons (3–5 PM). Cravings for sweet snacks have been reported to be more common in evenings than in the mornings [43]. However, our randomization procedure yielded approximately equal proportions of morning/afternoon participants in the two tDCS conditions, so we do not believe this would lead to any systematic differences in our findings. Finally, this study utilized a tDCS montage that others have used in the past (anode F4 (right), cathode F3 (left) [15]; however, this montage makes it difficult to understand whether cathodal or anodal stimulation of the left or right DLPFC, respectively, drive the results. Future work could examine this issue by placing cathodal electrode over ostensibly uninvolved brain regions (e.g., parietal regions) or by using an extracephalic reference electrode (e.g., over the deltoid). Despite these weaknesses, we believe this study contributes important observations about the effect of tDCS on food cravings.

## 5 Conclusions

Meta-analyses have shown non-invasive brain stimulation (rTMS and tDCS) targeting the DLPFC can influence cravings generally, and food cravings in particular. This well powered study found active tDCS reduced the perceived liking of food but did not reduce wanting. While tDCS has shown some promise, more work is needed to understanding how and when tDCS influences food cravings and consumption. Moreover, for tDCS to have impact beyond the lab, researchers will need to scale up laboratory studies into real world interventions that influence actual eating behavior in the daily life.

## Declarations

### Credit author statement

Eric Anderson; Julie Cantelon: Conceived and designed the experiments; Performed the experiments; Analyzed and interpreted the data; Contributed reagents, materials, analysis tools or data; Wrote the paper.Amanda Holmes: Performed the experiments.Grace Giles: Conceived and designed the experiments; Analyzed and interpreted the data.Tad Brunyé: Conceived and designed the experiments; Contributed reagents, materials, analysis tools or data; Wrote the paper.Robin Kanarek: Conceived and designed the experiments; Wrote the paper.

### Funding statement

This work was supported by the U.S. Army DEVCOM Soldier Center [W911-QY-13-R-0032] and the 10.13039/100006108National Center for Advancing Translational Sciences [Grant Number KL2TR002545].

### Data availability statement

Data will be made available on request.

## Ethics statement

The manuscript includes a statement that the study obtained ethics approval, including the name of the ethics committee(s) or institutional review board(s).

## Declaration of competing interest

The authors declare that they have no known competing financial interests or personal relationships that could have appeared to influence the work reported in this paper.
