# Supplementary material for: Transcranial direct current stimulation (tDCS) to dorsolateral prefrontal cortex influences perceived pleasantness of food
Source: Heliyon. 2023 Feb 4;9(2):e13275. doi: 10.1016/j.heliyon.2023.e13275 (PMC9929296; doi:10.1016/j.heliyon.2023.e13275)
Supplement: Multimedia component 1 [file mmc1.pdf]

PPID

EXPERIMENTER: Participant ID

Start

Before we begin the next task, you will complete a series of questionnaires. Please notify the experimenter if you have any questions.

Demographics

What is your current age?

What is your gender?

- Male
- Female
- Other

What is your height? (inches)

What is your weight? (lbs)

Do you have normal or corrected to normal vision?

- Yes

○ No

What is your ethnicity?

○ White
○ Black
○ Hispanic
○ Asian
○ Other

Is English your native language?

○ Yes
○ No

What is/was your college major?

[ ]

Are you on any birth control medication?

○ Yes
○ No

If yes, please indicate:

[ ]

Approximately how many days are there between the first day of one cycle and the first day of the next cycle?

[ ]

What is the date of the first day of your last period? YYYY-MM-DD

Edinburgh Handedness Inventory

Which of the following do you consider yourself to be?

- ○ Right-handed
- ○ Left-handed
- ○ Ambidextrous

Please indicate your preference in the use of hands for each of the following activities/objects.

	Always Left	Usually Left	No Preference	Usually Right	Always Right
Writing	○	○	○	○	○
Drawing	○	○	○	○	○
Throwing	○	○	○	○	○
Scissors	○	○	○	○	○
Toothbrush	○	○	○	○	○
Holding a knife to cut meat	○	○	○	○	○
Spoon	○	○	○	○	○
Broom (upper hand)	○	○	○	○	○
Striking a match	○	○	○	○	○
Opening a lid	○	○	○	○	○

Block 4

godin

During a typical 7-Day period (a week), how many times on average do you do the following kinds of exercise for more than 15 minutes during your free time (write on each line the appropriate number of hours per week).

STRENUOUS EXERCISE
(HEART BEATS RAPIDLY)
(e.g., running, jogging, hockey, football, soccer, squash, basketball, cross country skiing, judo, roller skating, vigorous swimming, vigorous long distance bicycling)

Enter Hours Per Week

MODERATE EXERCISE
(NOT EXHAUSTING)
(e.g., fast walking, baseball, tennis, easy bicycling, volleyball, badminton, easy swimming, alpine skiing, popular and folk dancing)

Enter Hours Per Week

MILD EXERCISE
(MINIMAL EFFORT)
(e.g., yoga, archery, fishing from river bank, bowling, horseshoes, golf, snow-mobiling, easy walking)

Enter Hours Per Week

During a typical 7-Day period (a week), in your leisure time, how often do you engage in the any regular activity long enough to work up a sweat (heart beats rapidly)?

- Often
- Sometimes
- Rarely/Never

Powered by Qualtrics

PPID

EXPERIMENTER: Participant ID

Start

Welcome! For the first part of this study you will be completing a series of questionnaires. Please notify the experimenter if you have any questions.

Screening Questions

Please note: You will not be able to participate in this study if you are a smoker, taking drugs/medications of any kind (other than oral contraceptives), pregnant or nursing, have a history of diabetes or food allergies.

Have you ever had an adverse reaction to tDCS (trans-cranial direct current stimulation)?

- Yes
- No

Have you ever had a seizure?

- Yes
- No

Have you ever had a head injury (including neurosurgery)?

- Yes
- No

Have you ever had any illness that caused brain injury?

○ Yes

○ No

Have you ever had any other brain-related condition?

○ Yes

○ No

Have you ever been diagnosed with a neurological or psychiatric disorder?

○ Yes

○ No

Do you have any metal in your head (outside of the mouth) such as shrapnel, surgical clips, or fragments from welding or metalwork?

○ Yes

○ No

Do you have a sensitive scalp (is your skin very dry, or do you use products designed for people with sensitive scalp)?

○ Yes

○ No

Have you participated in a study involving trans-cranial direct current stimulation within the past year?

○ Yes

○ No

Hunger Ratings

Please answer the following questions about your hunger and meals

Please answer the following questions about your hunger and meals

How hungry do you currently feel?

	not at all hungry				very hungry
	0	25	50	75	100
Drag the scale to display your opinion					

How many hours ago did you eat anything?

How many hours ago was your last full meal?

Eating Questionnaire Part 1/Albert J.Stunkar&Samuel Messick

Based on your personal views towards food, answer the following questions either true or false

	True	False
When I smell a sizzling steak or see a juicy piece of meat, I find it very difficult to keep from eating, even if I have just finished a meal.	○	○
I usually eat too much at social occasions, like parties and picnics.	○	○
I am usually so hungry that I eat more than three times a day.	○	○
When I have eaten my quota of calories, I am usually good about not eating any more.	○	○

	True	False
Dieting is so hard for me because I just get too hungry.	○	○
I deliberately take small helpings as a means of controlling my weight.	○	○

Sometimes things just taste so good that I keep on eating even when I am no longer hungry.

Since I am often hungry, I sometimes wish that while I am eating, an expert would tell me that I have had enough or that I can have something more to eat.

When I feel anxious, I find myself eating.

Life is too short to worry about dieting.

Since my weight goes up and down, I have gone on reducing diets more than once.

I often feel so hungry that I just have to eat something.

True False

When I am with someone who is overeating, I usually overeat too.

I have a pretty good idea of the number of calories in common food.

Sometimes when I start eating, I just can’t seem to stop.

It is not difficult for me to leave something on my plate.

At certain times of the day, I get hungry because I have gotten used to eating then

While on a diet, if I eat food that is not allowed, I consciously eat less for a period of time to make up for it

Being with someone who is eating often makes me hungry enough to eat also.

When I feel blue, I often overeat.

I enjoy eating too much to spoil it by counting calories or watching my weight.

When I see a real delicacy, I often get so hungry that I have to eat right away.

True False

I often stop eating when I am not really full as a conscious means of limiting the amount I eat.

I get so hungry that my stomach often seems like a bottomless pit.

True False

	True	False
My weight has hardly changed at all in the last ten years.	○	○
I am always hungry so it is hard for me to stop eating before I finish the food on my plate	○	○
When I feel lonely, I console myself by eating.	○	○
I consciously hold back at meals in order not to gain weight.	○	○
I sometimes get very hungry late in the evening or at night	○	○
I eat anything I want, any time I want.	○	○
Without even thinking about it, I take a long time to eat.	○	○
I count calories as a conscious means of controlling my weight.	○	○
I do not eat some foods because they make me fat.	○	○
I am always hungry enough to eat at any time.	○	○
I pay a great deal of attention to changes in my figure.	○	○
While on a diet, if I eat a food that is not allowed, I often then splurge and eat other high calorie foods	○	○

Eating Questionnaire Part 2/Albert J.Stunkar&Samuel Messick

Please answer the following questions by clicking the response that is appropriate to you.

How often are you dieting in a conscious effort to control your weight?

- ○ Rarely
- ○ Sometimes
- ○ Usually
- ○ Always

Would a weight fluctuation of 5 lbs affect the way you live your life?

- Not at all
- Slightly
- Moderately
- Very Much

How often do you feel hungry?

- Only At Meal Times
- Sometimes Between Meals
- Often Between Meals
- Almost Always

Do your feelings of guilt about overeating help you to control your food intake?

- Never
- Rarely
- Often
- Always

How difficult would it be for you to stop eating halfway through dinner and not eat for the next four hours?

- Easy
- Slightly Difficult
- Moderately Difficult
- Very Difficult

How conscious are you of what you are eating?

- Not At All
- Slightly
- Moderately
- [illegible]

- Extremely

How frequently do you avoid ‘stocking up’ on tempting foods?

- Almost Never
- Seldom
- Usually
- Almost Always

How likely are you to shop for low calorie foods?

- Unlikely
- Slightly Unlikely
- Moderately Likely
- Very Likely

Do you eat sensibly in front of others and splurge alone?

- Never
- Rarely
- Often
- Always

How likely are you to consciously eat slowly in order to cut down on how much YOU eat?

- Unlikely
- Slightly Likely
- Moderately Likely
- Very Likely

How frequently do you skip dessert because you are no longer hungry?

- Almost Never
- Seldom
- At Least Once A Week

- At Least Once A Week
- Almost Every Day

How likely are you to consciously eat less than you want?

- Extremely likely
- Moderately likely
- Slightly likely
- Neither likely nor unlikely
- Slightly unlikely
- Moderately unlikely
- Extremely unlikely

Do you go on eating binges though you are not hungry?

- Definitely yes
- Probably yes
- Might or might not
- Probably not
- Definitely not

On a scale of 0 to 5, where 0 means no restraint in eating (eating whatever you want, whenever you want it) and 5 means total restraint (constantly limiting food intake and never ‘giving in’), what number would you give yourself?

- 0 : eat whatever you want, whenever you want it
- 1 : usually eat whatever you want, whenever you want it
- 2 : often eat whatever you want, whenever you want it
- 3 : often limit food intake, but often ‘give in’
- 4 : usually limit food intake, rarely ‘give in’
- 5 : constantly limiting food intake, never ‘giving in’

To what extent does this statement describe your eating behavior ? ‘I start dieting in the morning, but because of any number of things that happen during the day, by evening I

have given up and eat what I want, promising myself to start dieting again tomorrow.'

- Not like me
- Little Like Me
- Pretty Good Description of Me
- Describes Me Perfectly

FCQ-Trait(Nijs)

Indicate how often each statement would be true for you, in general:

	1<br>never (or not applicable)	2<br>rarely	3<br>sometimes	4<br>often	5<br>usually	6<br>always
I feel like I have food on my mind all the time						
I can't stop thinking about eating no matter how hard I try						
I find myself preoccupied with food						
If I am craving something, thoughts of eating it consume me						

	1<br>never (or not applicable)	2<br>rarely	3<br>sometimes	4<br>often	5<br>usually	6<br>always
Food cravings invariably make me think of ways to get what I want to eat						
I spend a lot of time thinking about whatever it is I will eat next						
If I eat what I'm craving, I often lose control and eat too much						
Once I start eating, I have trouble stopping						
[illegible]						

When I crave something, I know I won't be able to stop eating once I start	O	O	O	O	O	O
If I get what I'm craving I cannot stop myself from eating it	O	O	O	O	O	O
When I am with someone who is overeating, I usually overeat too	O	O	O	O	O	O
Whenever I go to a buffet I end up eating more than what I needed	O	O	O	O	O	O
	never (or not applicable)	rarely	sometimes	often	usually	always

	never (or not applicable)	rarely	sometimes	often	usually	always
Eating what I crave makes me feel better	O	O	O	O	O	O
When I eat what I crave, I feel great	O	O	O	O	O	O
I feel less anxious after I eat	O	O	O	O	O	O
When I eat food, I feel comforted	O	O	O	O	O	O
Sometimes, eating makes things seem just perfect	O	O	O	O	O	O
I crave foods when I'm upset	O	O	O	O	O	O

	1	2	3	4	5	6
	never (or not applicable)	rarely	sometimes	often	usually	always
My emotions often make me want to eat	O	O	O	O	O	O
When I'm stressed out, I crave food	O	O	O	O	O	O
I crave foods when I feel bored, angry or sad	O	O	O	O	O	O

FCQ-State(Nijs)

Indicate the extent to which you agree with each statement right now, at this very moment:

	1	2	3	4	5

	strongly disagree	disagree	neutral	agree	strongly agree
I'm craving tasty food	○	○	○	○	○
I have an urge for tasty food	○	○	○	○	○
I have an intense desire to eat something tasty	○	○	○	○	○
If I ate something, I wouldn't feel so sluggish and lethargic	○	○	○	○	○
Satisfying my appetite would make me feel less grouchy and irritable	○	○	○	○	○
I would feel more alert if I could satisfy my appetite	○	○	○	○	○
If I ate right now, my stomach wouldn't feel as empty	○	○	○	○	○
I am hungry	○	○	○	○	○
I feel weak because of not eating	○	○	○	○	○
My desire to eat something tasty seems overpowering	○	○	○	○	○

	1<br>strongly disagree	2<br>disagree	3<br>neutral	4<br>agree	5<br>strongly agree
I know I'm going to keep on thinking about tasty food until I actually have it	○	○	○	○	○
If I had something tasty to eat, I could not stop eating it	○	○	○	○	○
If I were to eat what I'm desiring, I am sure my mood would improve	○	○	○	○	○
Eating something tasty would feel wonderful	○	○	○	○	○
Eating something tasty would make things just perfect	○	○	○	○	○
	strongly disagree	disagree	neutral	agree	strongly agree

Measure of Motives and Ethics (FCQ)/ Steptoe, Pollard, Wardle

Report how important the following statements are.

It is important to me that the food I eat on a typical day:

	1<br>not at all important	2<br>a little important	3<br>moderately important	4<br>very important
Contains a lot of vitamins and minerals	○	○	○	○
Keeps me healthy	○	○	○	○
Is nutritious	○	○	○	○
Is high in protein	○	○	○	○
Is good for my skin/teeth/hair/nails etc	○	○	○	○
Is high in fibre and roughage	○	○	○	○
Helps me cope with stress	○	○	○	○
Helps me to cope with life	○	○	○	○

	1<br>not at all important	2<br>a little important	3<br>moderately important	4<br>very important
Helps me relax	○	○	○	○
Keeps me awake/alert	○	○	○	○
Cheers me up	○	○	○	○
Makes me feel good	○	○	○	○

	not at all important	a little important	moderately important	very important
Is easy to prepare	○	○	○	○
Can be cooked very simply	○	○	○	○
Takes no time to prepare	○	○	○	○
Can be bought in shops close to where I live or work	○	○	○	○
Is easily available in shops and supermarkets	○	○	○	○
Smells nice	○	○	○	○

Looks nice	○	○	○	○
Has a pleasant texture	○	○	○	○
Tastes good	○	○	○	○
Contains no additives	○	○	○	○
Contains natural ingredients	○	○	○	○
Contains no artificial ingredients	○	○	○	○
	not at all important	a little important	moderately important	very important

Is not expensive	○	○	○	○
Is cheap	○	○	○	○
Is good value for money	○	○	○	○
Is low in calories	○	○	○	○
Helps me control my weight	○	○	○	○
Is low in fat	○	○	○	○
Is what I usually eat	○	○	○	○

	1	2	3	4
	not at all important	a little important	moderately important	very important
Is familiar	○	○	○	○
Is like the food I ate when I was a child	○	○	○	○
Comes from countries I approve of politically	○	○	○	○
Has the country of origin clearly marked	○	○	○	○
Is packaged in an environmentally friendly way	○	○	○	○

Ethics/Lindeman&Vaananen

It is important that the food I eat on a typical day:

	1	2	3	4
	not at all important	slightly important	moderately important	very important

Has been produced in a way that

Has been produced in a way that animals have not experienced pain	○	○	○	○
Has been produced in a way that animals' rights have been respected	○	○	○	○
Has been prepared in an environmentally friendly way	○	○	○	○
Has been produced in a way which has not shaken the balance of nature	○	○	○	○
Is packaged in an environmentally friendly way	○	○	○	○
Comes from a country I approve of politically	○	○	○	○
Comes from a country in which humans rights are not violated	○	○	○	○
Has the country of origin clearly marked	○	○	○	○

	1<br>not at all important	2<br>slightly important	3<br>moderately important	4<br>very important
Has been prepared in a way that does not conflict with my political vaules	○	○	○	○
Is not forbidden in my religion	○	○	○	○
Is in harmony with my religious views	○	○	○	○

Block 10

Powered by Qualtrics

Start Program

Please wait for the experimenter to start the program. Thank you!

PPID

EXPERIMENTER: Participant ID

Block 3

For the first part of this study you will be completing a series of questions based on social cravings. Please notify the experimenter if you have any questions.

Social Cravings

People often crave connecting with other people, including friends and family. People connect socially in person, or using phones, or social networking platforms. Social connection could include calling your mom, texting a friend, posting an update on Facebook, or checking Twitter. For the following questions, consider any of these methods of connecting--as well as any other ways you personally connect with people.

	1<br>Strongly Disagree	2<br>Somewhat Disagree	3<br>Neither Agree Nor Disagree	4<br>Somewhat Agree	5<br>Strongly Agree
I’m craving social connection	○	○	○	○	○
I have an urge for social connection	○	○	○	○	○
I have an intense desire to connect with other people	○	○	○	○	○

	1	2	3	4	5
			[illegible]		

	Strongly Disagree	Somewhat Disagree	Neither Agree Nor Disagree	Somewhat Agree	Strongly Agree
If I posted something, I wouldn’t feel so sluggish and lethargic	O	O	O	O	O
Connecting with people would make me feel less grouchy and irritable	O	O	O	O	O
I would feel more alert if I could connect socially	O	O	O	O	O
If I connected right now, I wouldn't feel as alone	O	O	O	O	O
I am alone	O	O	O	O	O
I feel alone because of a lack of connection	O	O	O	O	O
My desire to connect with people seems overpowering	O	O	O	O	O
I know I’m going to keep on thinking about connecting with people until I actually do it	O	O	O	O	O
If I started connecting with people, I could not stop it	O	O	O	O	O
If I were to connect in the way I’m desiring, I am sure my mood would improve	O	O	O	O	O
Connecting with people would feel wonderful	O	O	O	O	O
Connecting with people would make things just perfect	O	O	O	O	O

Start Program

Please wait for the experimenter to start the program. Thank you!

PPID

EXPERIMENTER: Participant ID (add "_PRE" or "_POST")

Instructions

For the following images, please rate how pleasant it would be to eat these foods, and how much you'd like to eat them. There is no right or wrong answer. For each image, imagine that the food is right in front of you and go with you gut reaction. Also, you need to move the cursor for your response to be logged--if you don't move it, no response is recorded. If you want the middle, move it away and back.

0004

0005

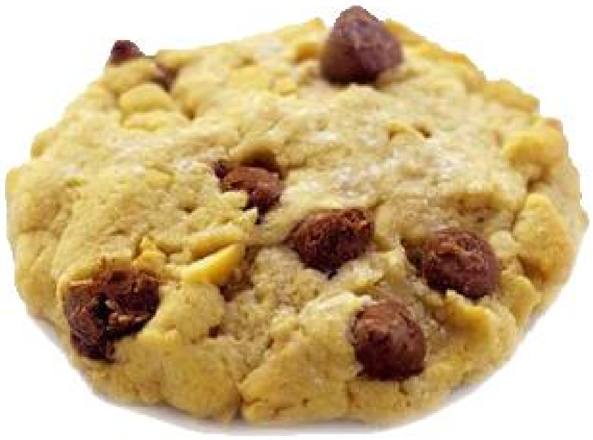

Very unpleasant | Very pleasant
1 | 100

How pleasant would it be to eat this?

No urge to eat | Extremely strong urge to eat
1 | 100

How strong is your urge to eat this?

0005

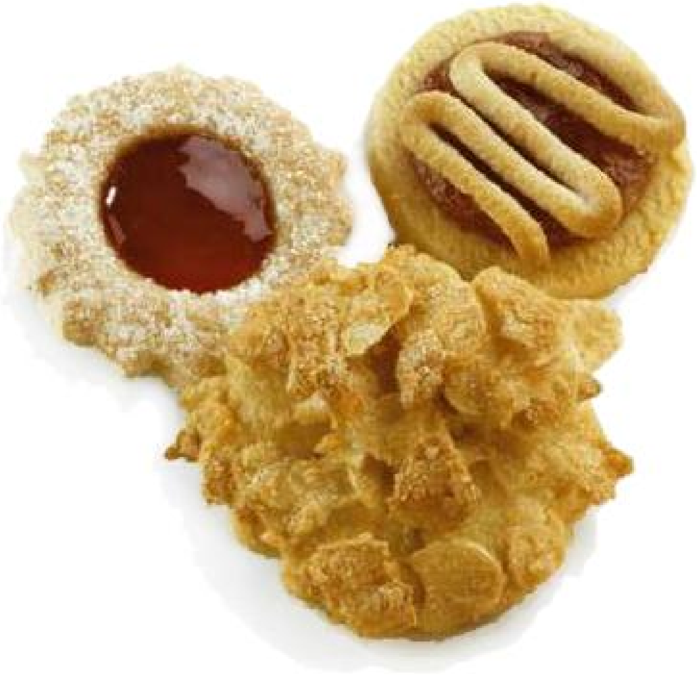

Very Unpleasant

Very Pleasant

1

100

How pleasant would it be to eat this?

No urge to eat

Extremely strong urge to eat

1

100

How strong is your urge to eat this?

0009

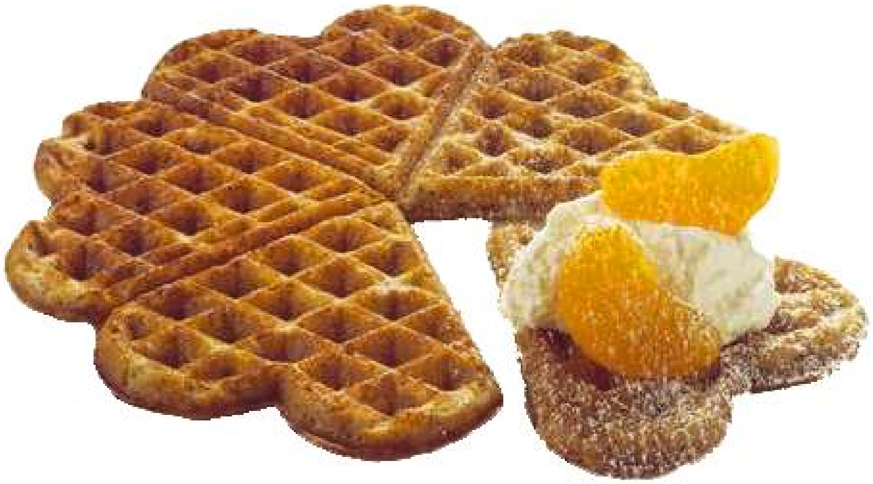

Very Unpleasant

Very Pleasant

1

100

How pleasant would it be to eat this?

No urge to eat

Extremely strong urge to eat

1

100

How strong is your urge to eat this?

0016

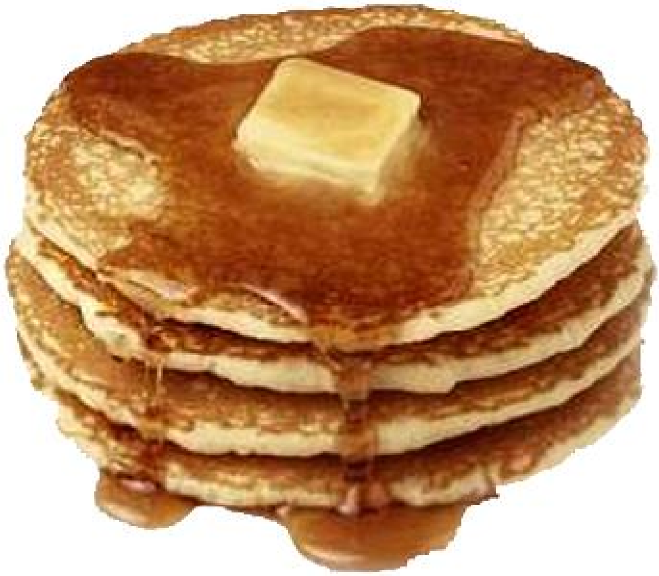

Very Unpleasant | Very Pleasant

1 | 100

How pleasant would it be to eat this?

No urge to eat | Extremely strong urge to eat

1 | 100

How strong is your urge to eat this?

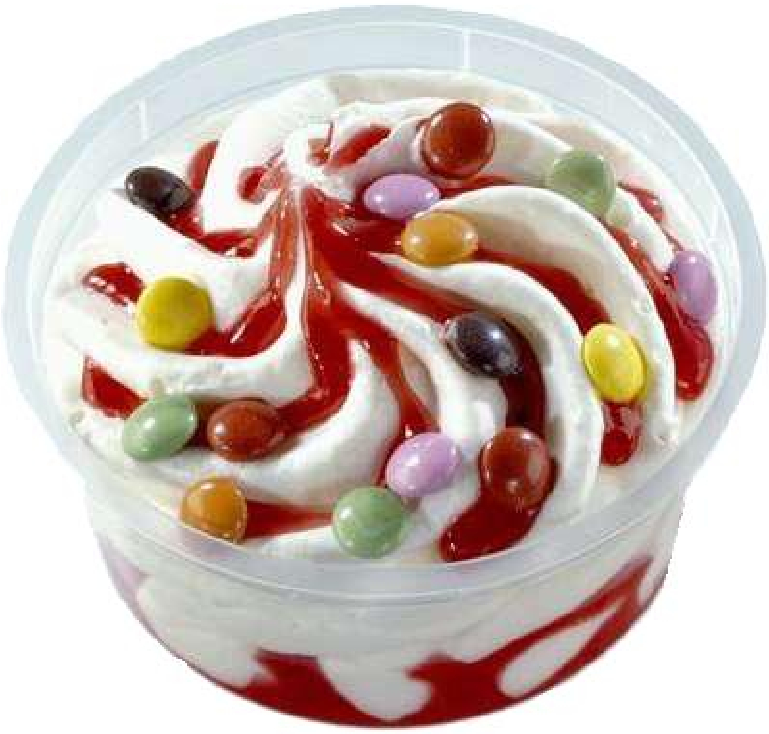

Very Unpleasant | Very Pleasant

1 | 100

How pleasant would it be to eat this?

No urge to eat | Extremely strong urge to eat

1 | 100

How strong is your urge to eat this?

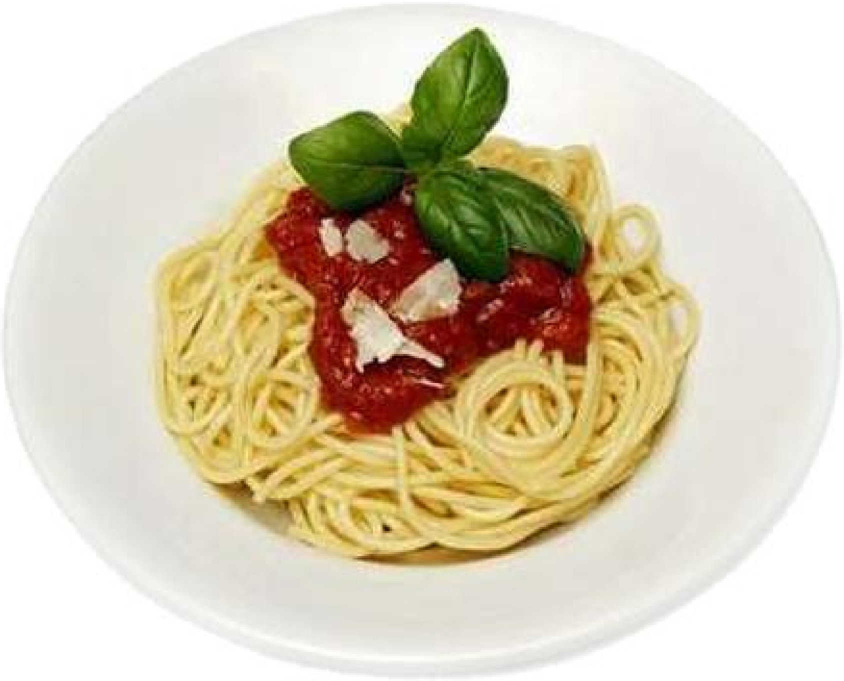

Very Unpleasant

Very Pleasant

1

100

How pleasant would
it be to eat this?

No urge to eat

Extremely strong
urge to eat

1

100

How strong is your
urge to eat this?

0036

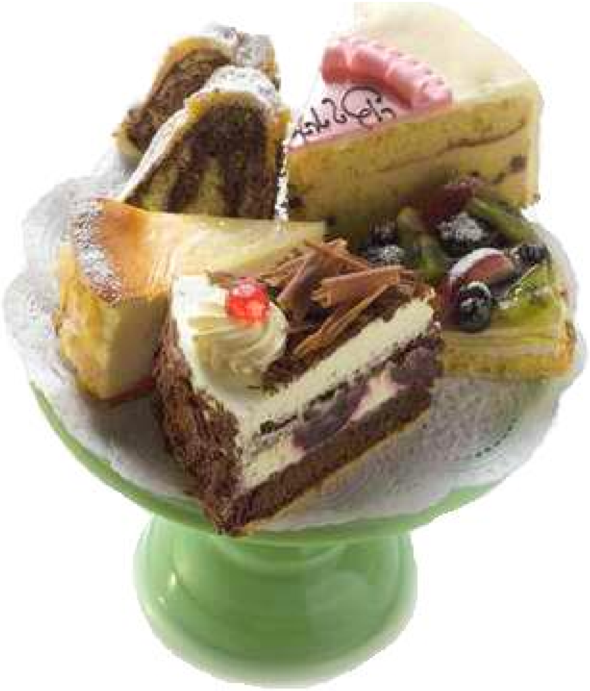

Very Unpleasant

Very Pleasant

1

100

How pleasant would
it be to eat this?

No urge to eat

Extremely strong
urge to eat

1

100

How strong is your
urge to eat this?

0046

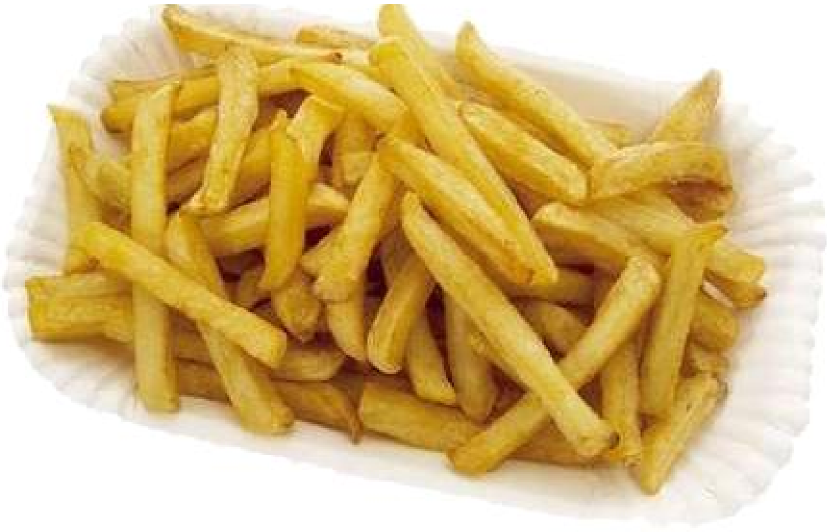

Very Unpleasant

Very Pleasant

1

100

How pleasant would it be to eat this?

No urge to eat

Extremely strong urge to eat

1

100

How strong is your urge to eat this?

0048

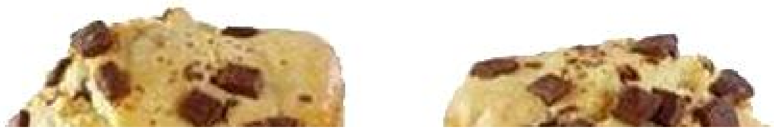

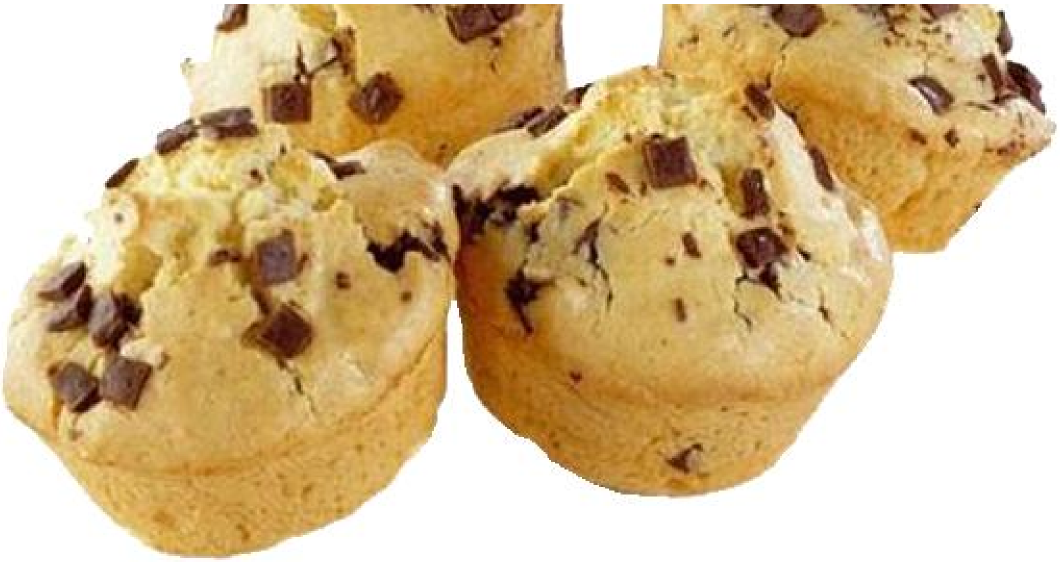

Very Unpleasant

Very Pleasant

1

100

How pleasant would it be to eat this?

No urge to eat

Extremely strong urge to eat

1

100

How strong is your urge to eat this?

0050

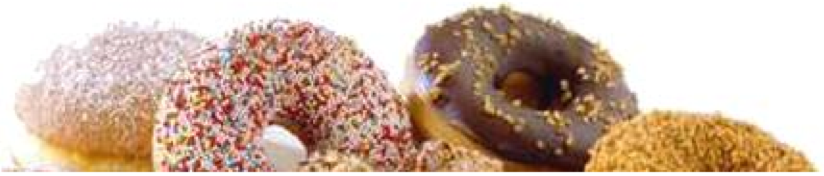

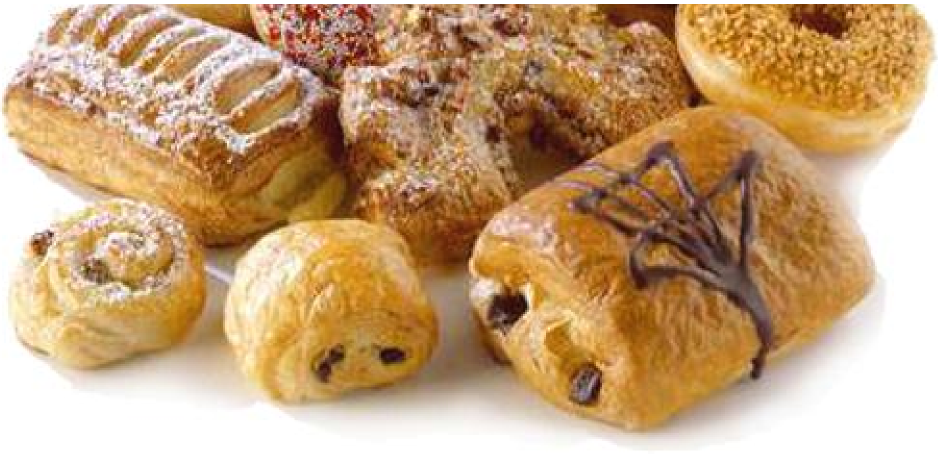

Very Unpleasant

Very Pleasant

1

100

How pleasant would it be to eat this?

No urge to eat

Extremely strong urge to eat

1

100

How strong is your urge to eat this?

0054

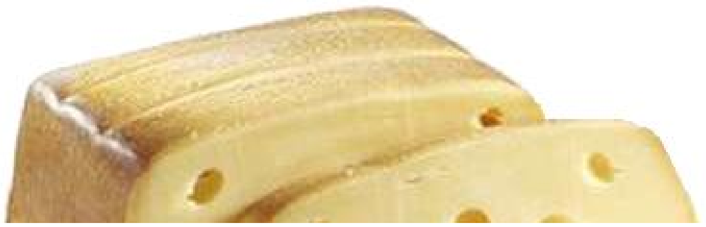

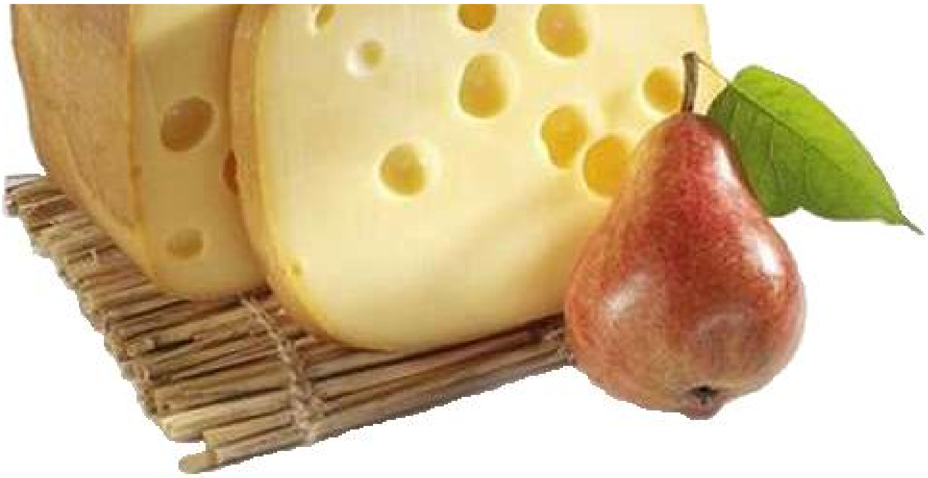

Very Unpleasant

Very Pleasant

1

100

How pleasant would it be to eat this?

No urge to eat

Extremely strong urge to eat

1

100

How strong is your urge to eat this?

0056

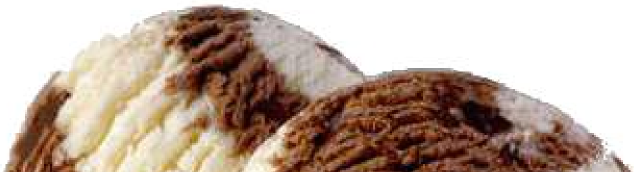

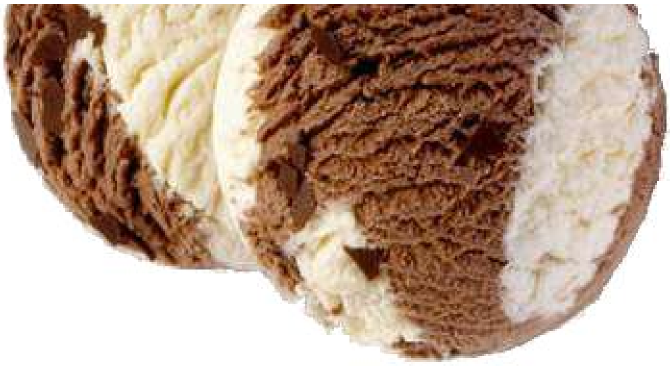

Very Unpleasant

Very Pleasant

1

100

How pleasant would
it be to eat this?

No urge to eat

Extremely strong
urge to eat

1

100

How strong is your
urge to eat this?

0074

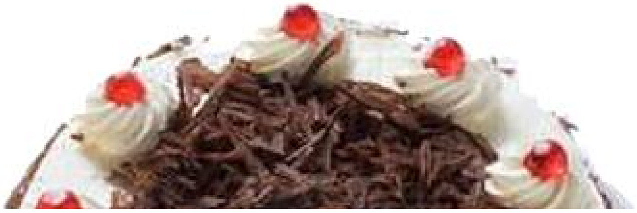

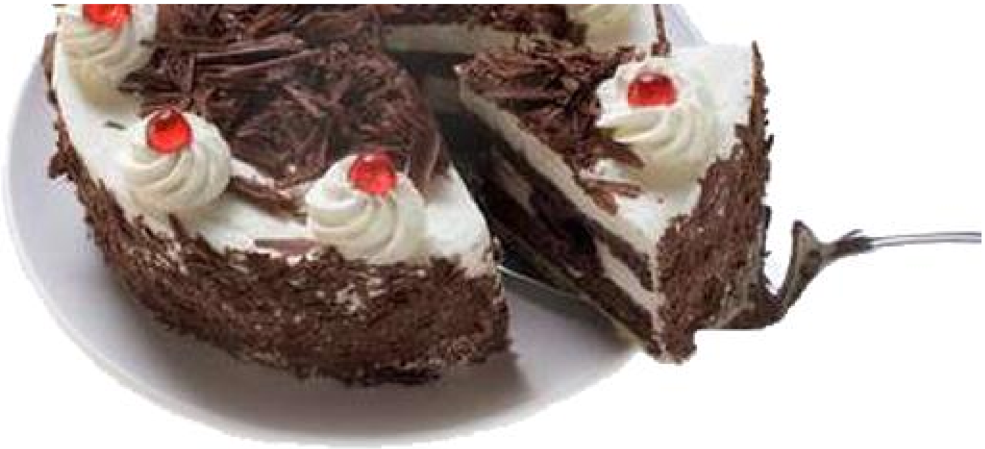

Very Unpleasant

Very Pleasant

1

100

How pleasant would it be to eat this?

No urge to eat

Extremely strong urge to eat

1

100

How strong is your urge to eat this?

0082

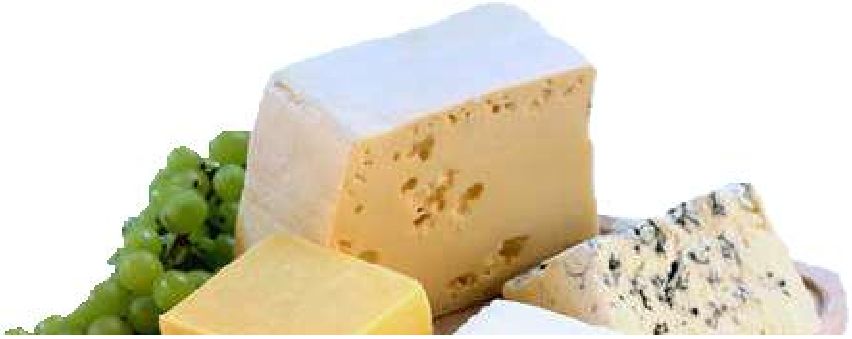

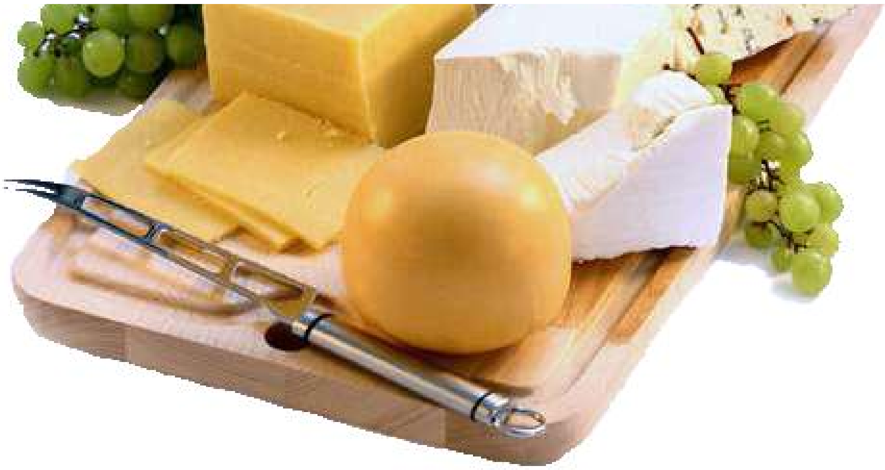

Very Unpleasant

Very Pleasant

1

100

How pleasant would it be to eat this?

No urge to eat

Extremely strong urge to eat

1

100

How strong is your urge to eat this?

0089

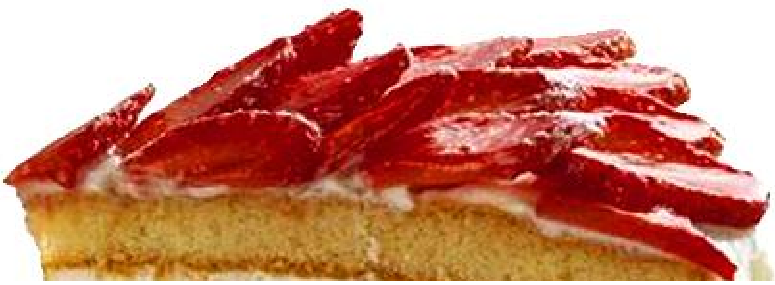

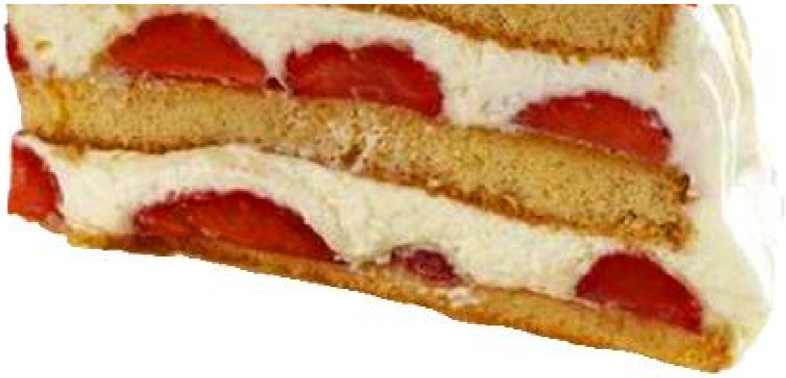

Very Unpleasant | Very Pleasant
1 | 100

How pleasant would it be to eat this?

No urge to eat | Extremely strong urge to eat
1 | 100

How strong is your urge to eat this?

0106

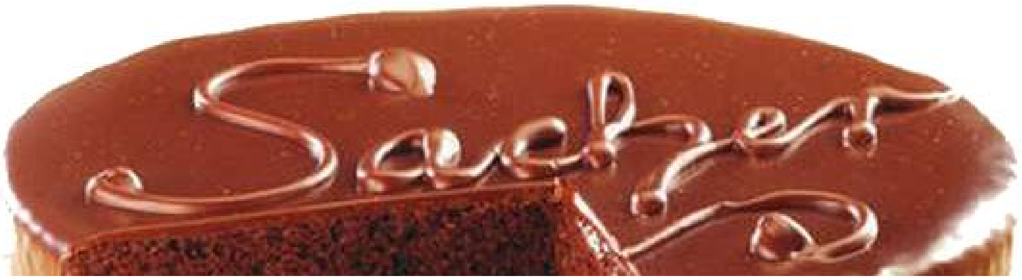

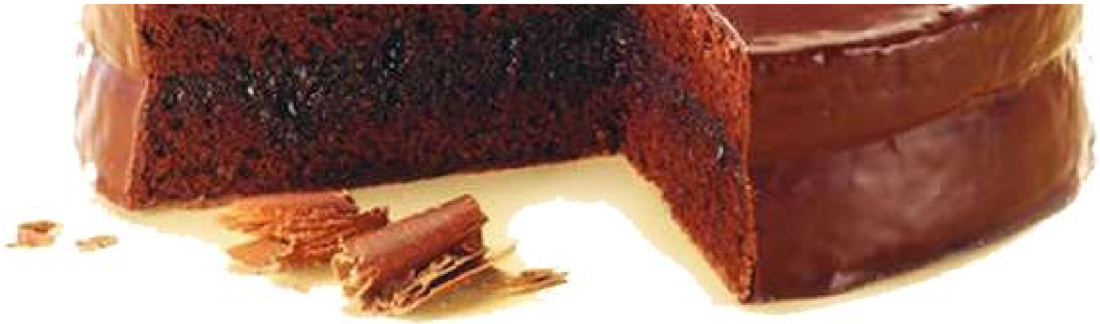

Very Unpleasant

Very Pleasant

1

100

How pleasant would
it be to eat this?

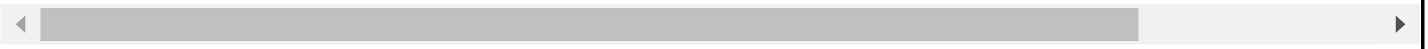

No urge to eat

Extremely strong
urge to eat

1

100

How strong is your
urge to eat this?

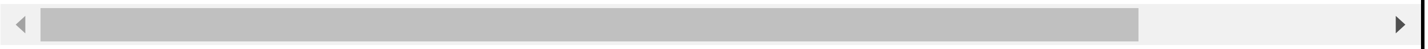

0107

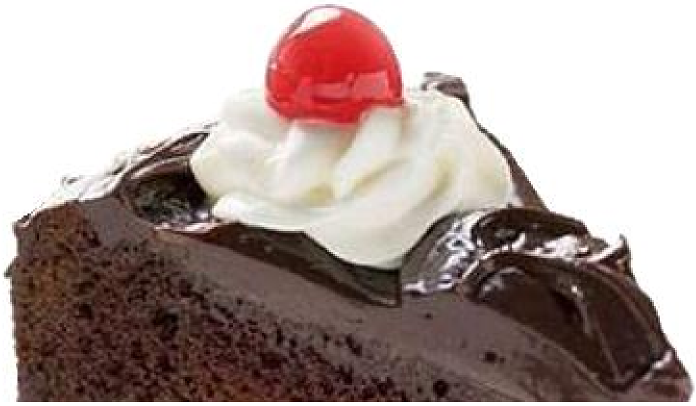

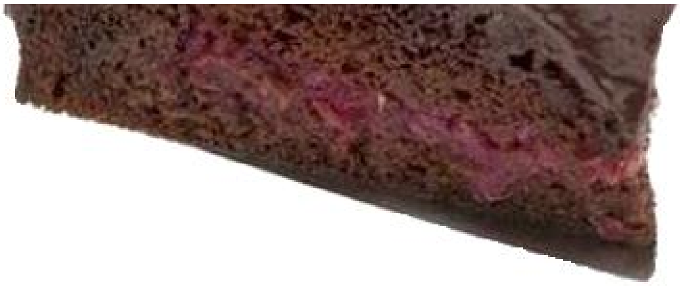

Very Unpleasant | Very Pleasant

1 | 100

How pleasant would it be to eat this?

No urge to eat | Extremely strong urge to eat

1 | 100

How strong is your urge to eat this?

0111

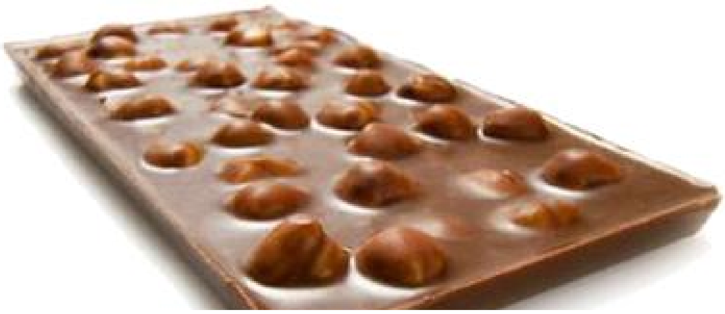

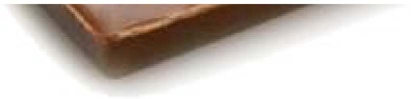

Very Unpleasant

Very Pleasant

1

100

How pleasant would it be to eat this?

No urge to eat

Extremely strong urge to eat

1

100

How strong is your urge to eat this?

0112

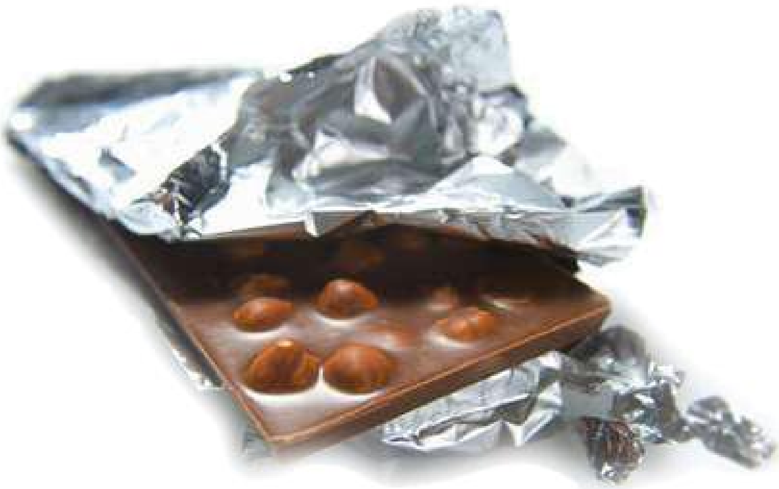

Very Unpleasant

Very Pleasant

1

100

How pleasant would it be to eat this?

No urge to eat

Extremely strong urge to eat

1

100

How strong is your urge to eat this?

1113

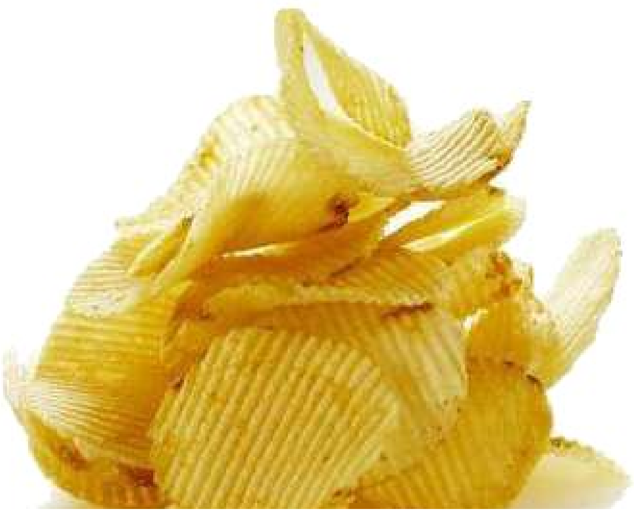

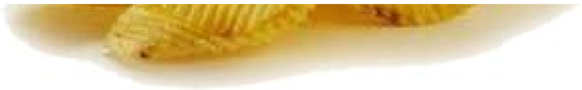

Very Unpleasant
Very Pleasant

1
100

How pleasant would it be to eat this?

No urge to eat
Extremely strong urge to eat

1
100

How strong is your urge to eat this?

0114

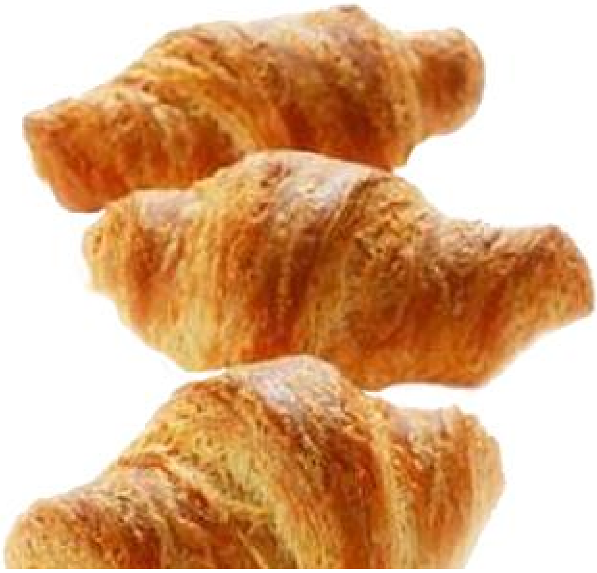

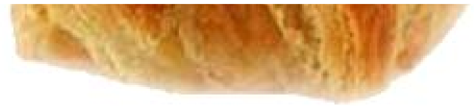

Very Unpleasant

Very Pleasant

1

100

How pleasant would it be to eat this?

No urge to eat

Extremely strong urge to eat

1

100

How strong is your urge to eat this?

0115

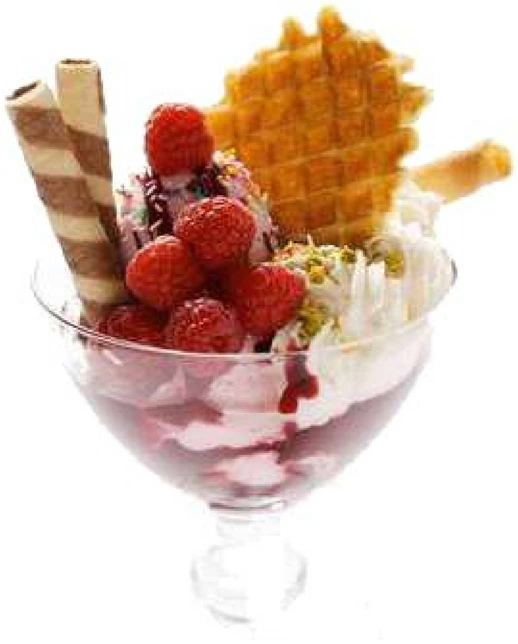

Very Unpleasant

Very Pleasant

1

100

How pleasant would it be to eat this?

No urge to eat

Extremely strong urge to eat

1

100

How strong is your urge to eat this?

0169

0131

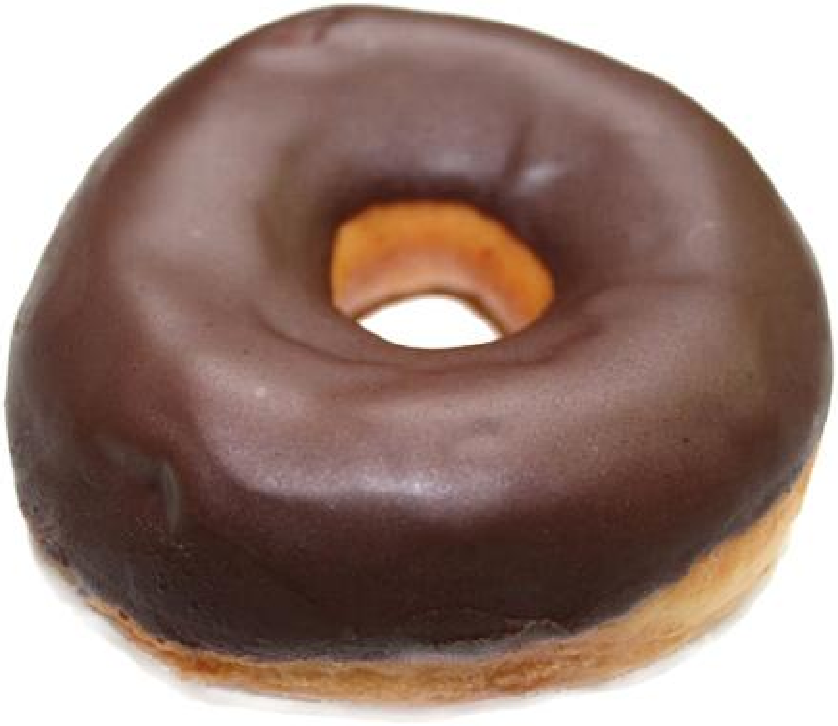

Very Unpleasant

Very Pleasant

1

100

How pleasant would it be to eat this?

No urge to eat

Extremely strong urge to eat

1

100

How strong is your urge to eat this?

0131

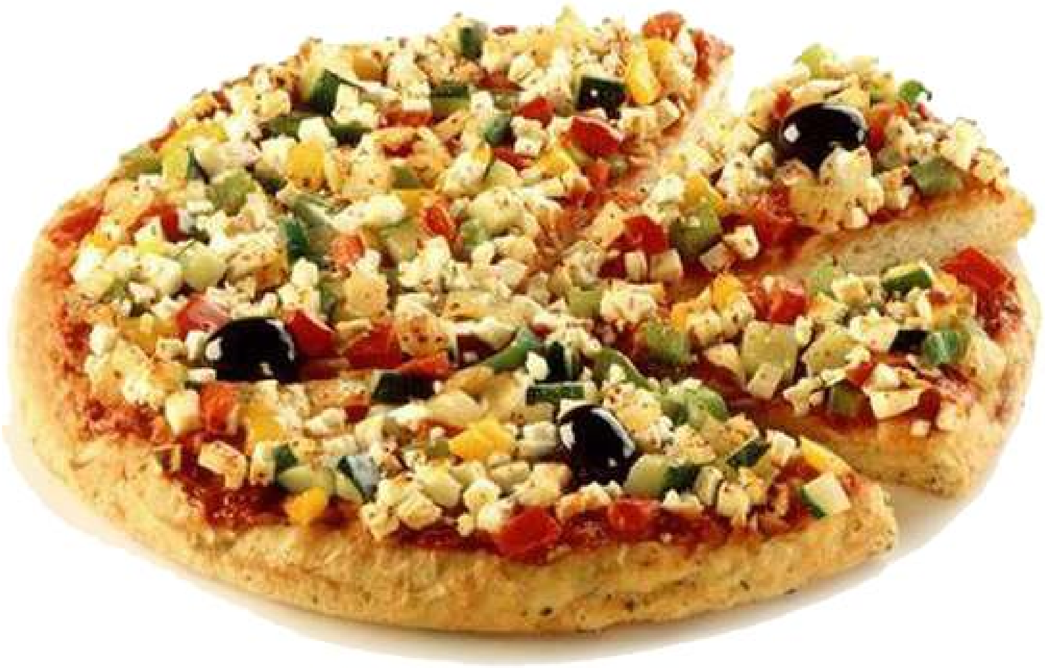

Very Unpleasant

Very Pleasant

1

100

How pleasant would it be to eat this?

No urge to eat

Extremely strong urge to eat

1

100

How strong is your urge to eat this?

0141

0173

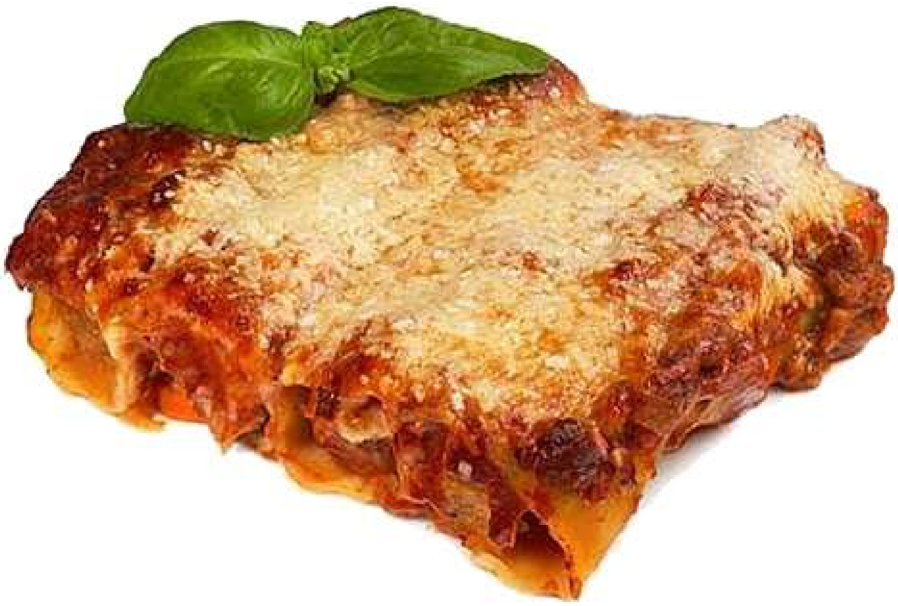

Very Unpleasant

1

Very Pleasant

100

How pleasant would it be to eat this?

No urge to eat

1

Extremely strong urge to eat

100

How strong is your urge to eat this?

0173

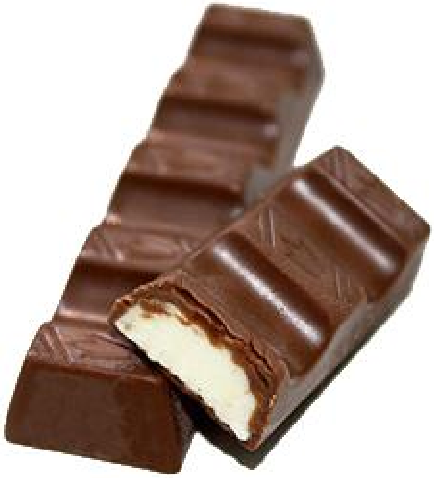

Very Unpleasant

Very Pleasant

1

100

How pleasant would
it be to eat this?

No urge to eat

Extremely strong
urge to eat

1

100

How strong is your
urge to eat this?

0206

0209

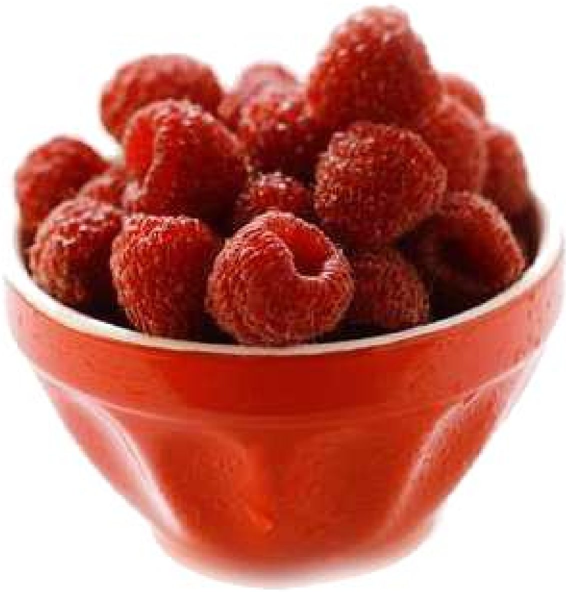

Very Unpleasant | Very Pleasant

1 | 100

How pleasant would it be to eat this?

No urge to eat | Extremely strong urge to eat

1 | 100

How strong is your urge to eat this?

0209

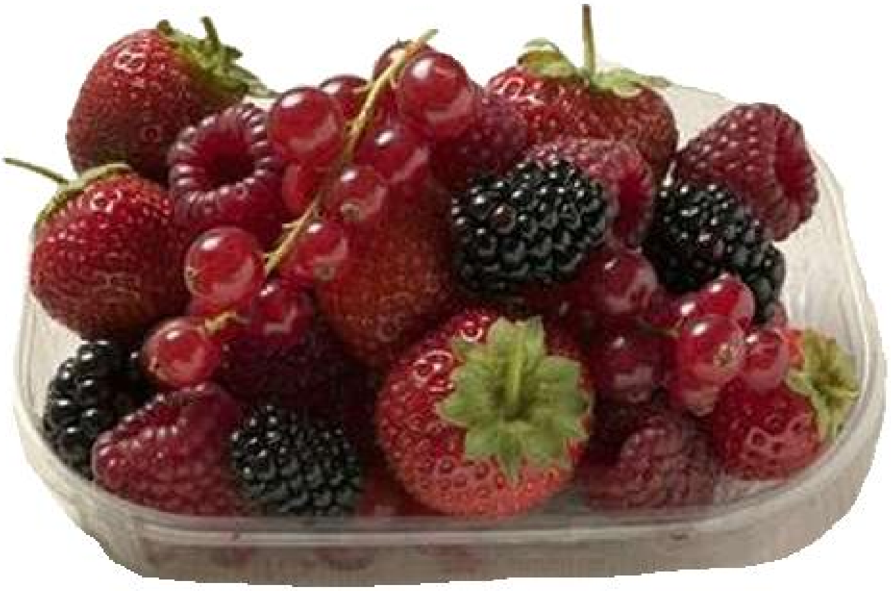

Very Unpleasant

Very Pleasant

1

100

How pleasant would
it be to eat this?

No urge to eat

Extremely strong
urge to eat

1

100

How strong is your
urge to eat this?

0211

0217

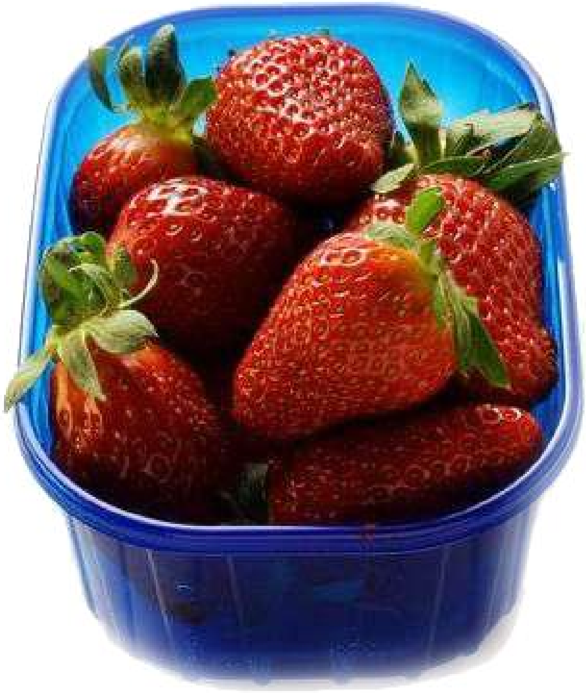

Very Unpleasant | Very Pleasant
1 | 100

How pleasant would it be to eat this?

No urge to eat | Extremely strong urge to eat
1 | 100

How strong is your urge to eat this?

0217

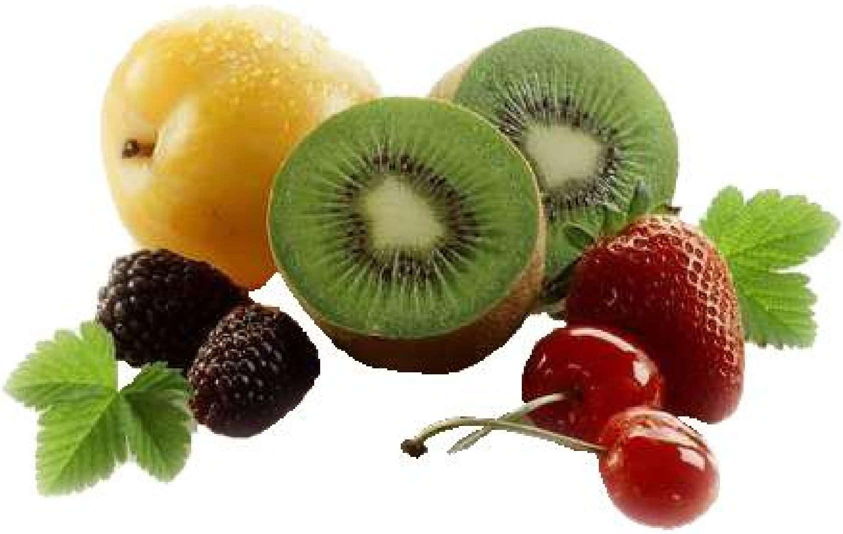

Very Unpleasant

Very Pleasant

1

100

How pleasant would it be to eat this?

No urge to eat

Extremely strong urge to eat

1

100

How strong is your urge to eat this?

0220

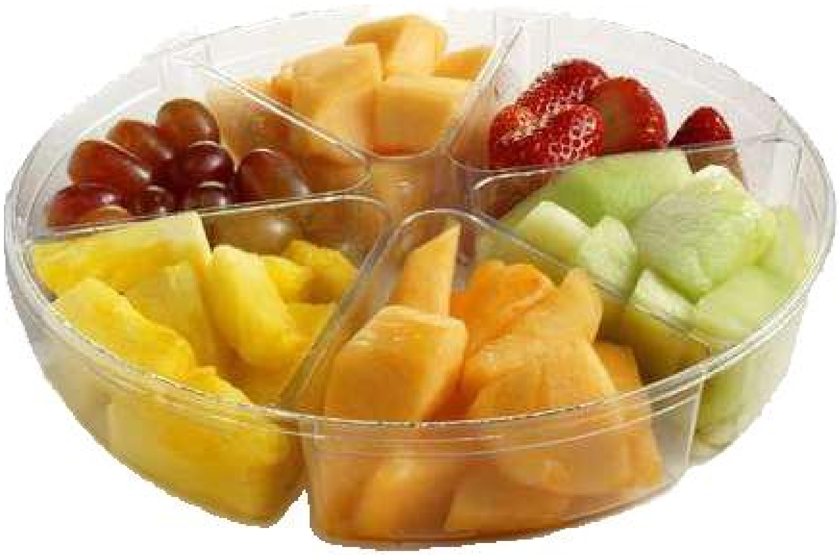

Very Unpleasant

Very Pleasant

1

100

How pleasant would it be to eat this?

No urge to eat

Extremely strong urge to eat

1

100

How strong is your urge to eat this?

0291

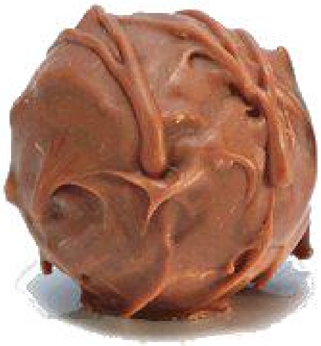

Very Unpleasant

Very Pleasant

1

100

How pleasant would it be to eat this?

No urge to eat

Extremely strong urge to eat

1

100

How strong is your urge to eat this?

0304

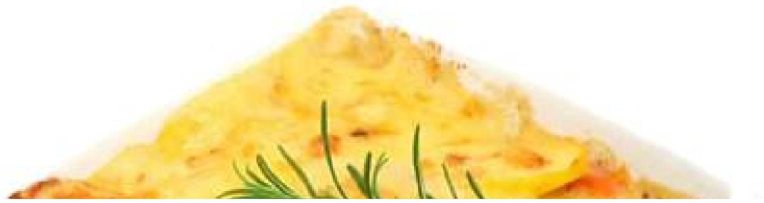

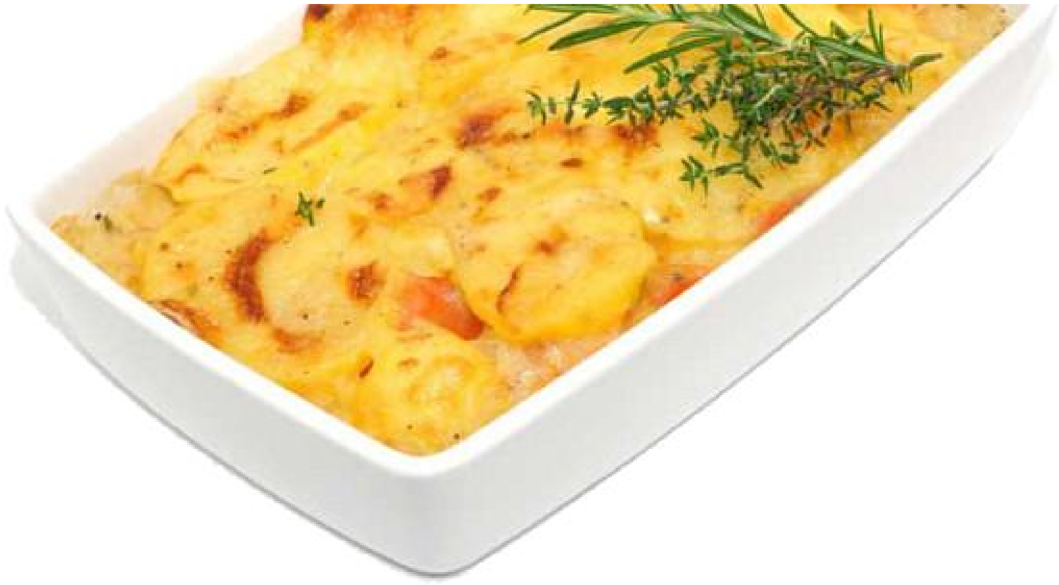

Very Unpleasant

Very Pleasant

1

100

How pleasant would it be to eat this?

No urge to eat

Extremely strong urge to eat

1

100

How strong is your urge to eat this?

0325

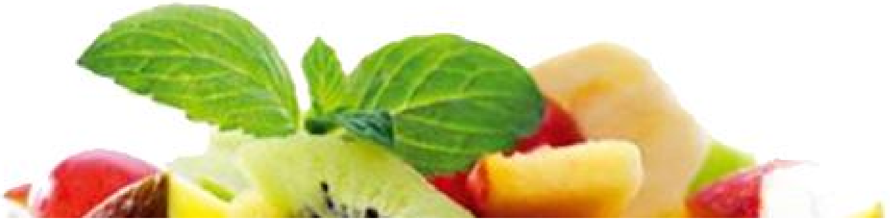

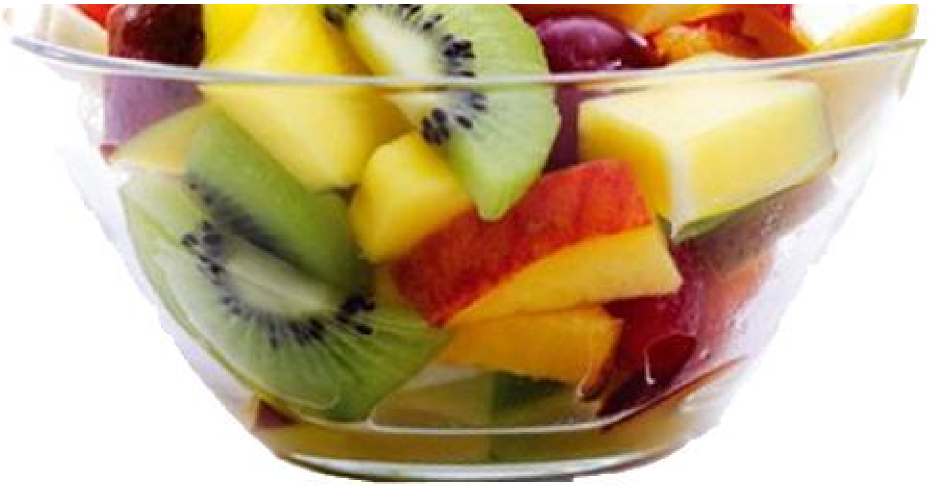

Very Unpleasant

Very Pleasant

1

100

How pleasant would it be to eat this?

No urge to eat

Extremely strong urge to eat

1

100

How strong is your urge to eat this?

0363

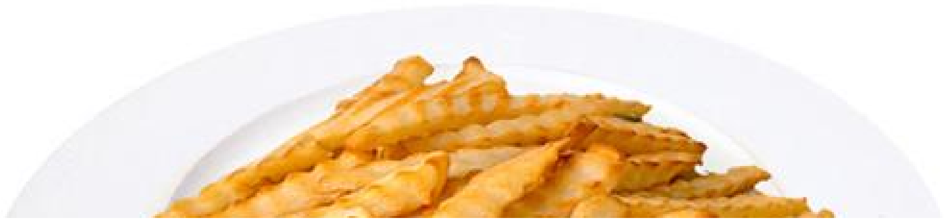

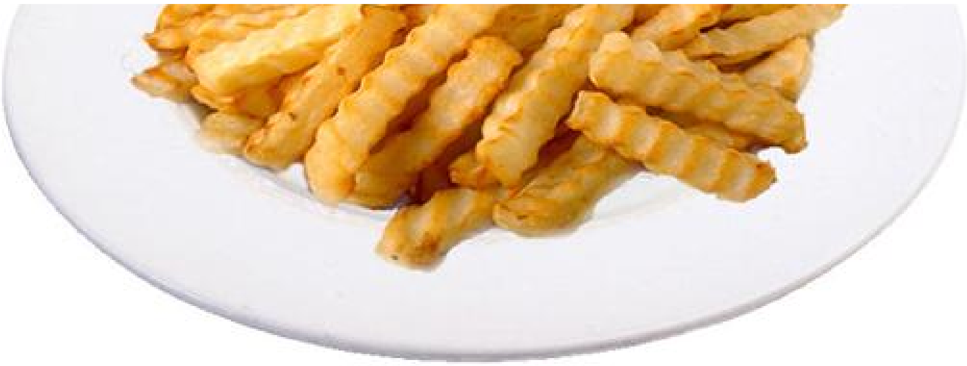

Very Unpleasant

Very Pleasant

1

100

How pleasant would it be to eat this?

No urge to eat

Extremely strong urge to eat

1

100

How strong is your urge to eat this?

0394

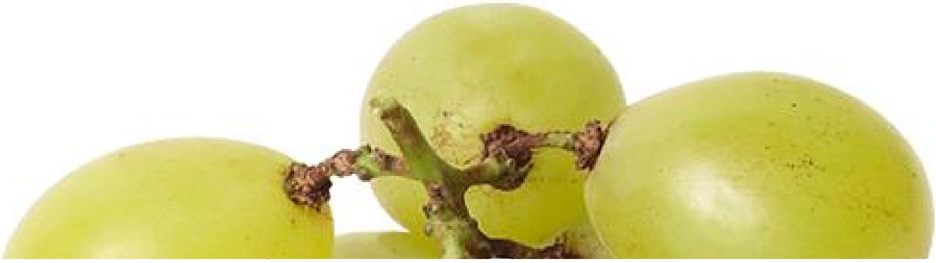

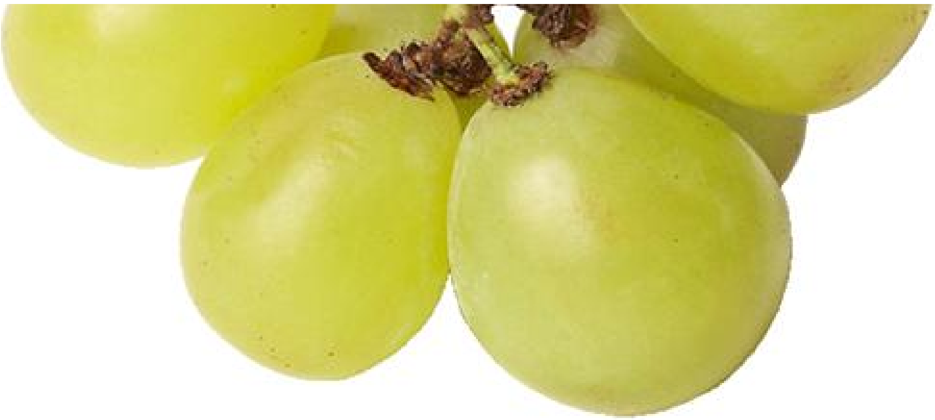

Very Unpleasant

Very Pleasant

1

100

How pleasant would it be to eat this?

No urge to eat

Extremely strong urge to eat

1

100

How strong is your urge to eat this?

0398

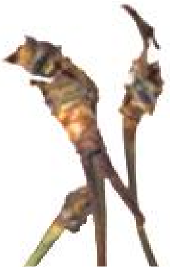

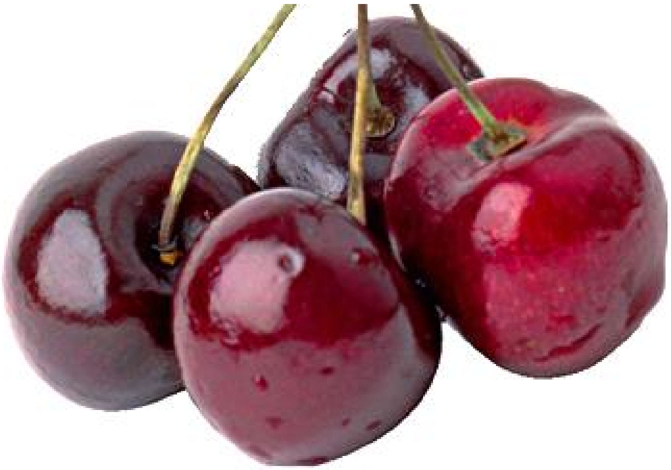

Very Unpleasant | Very Pleasant
1 | 100

How pleasant would it be to eat this?

No urge to eat | Extremely strong urge to eat
1 | 100

How strong is your urge to eat this?

0416

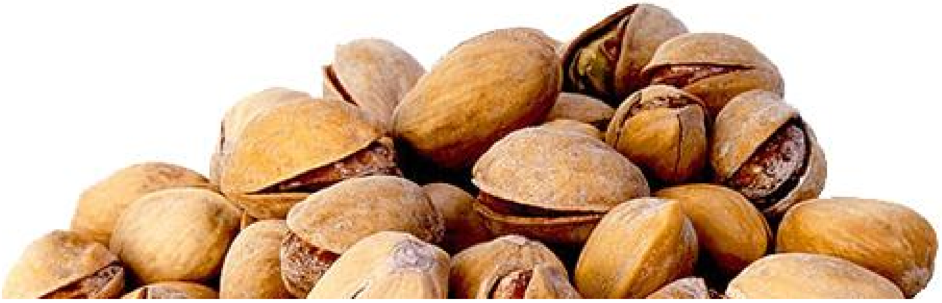

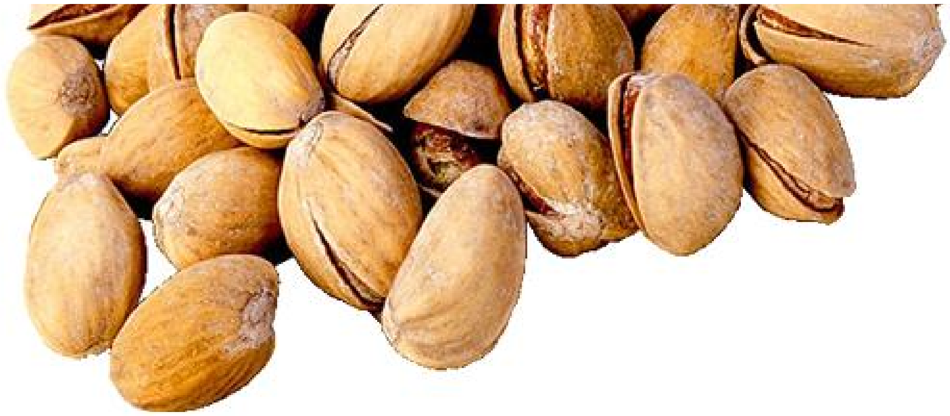

Very Unpleasant

Very Pleasant

1

100

How pleasant would it be to eat this?

No urge to eat

Extremely strong urge to eat

1

100

How strong is your urge to eat this?

0472

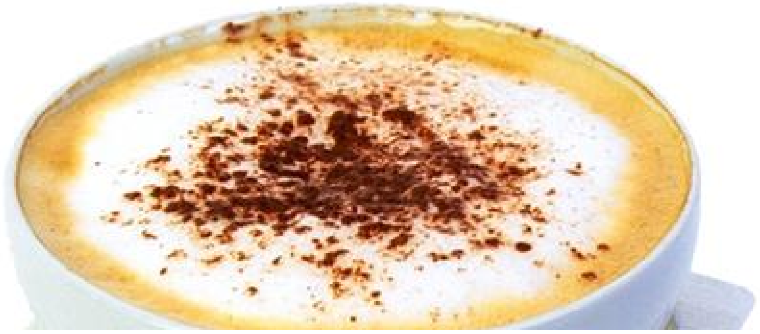

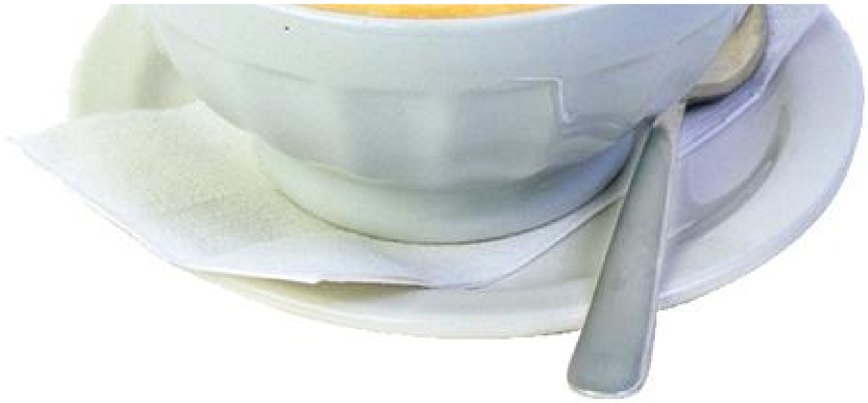

Very Unpleasant | Very Pleasant
1 | 100

How pleasant would it be to eat this?

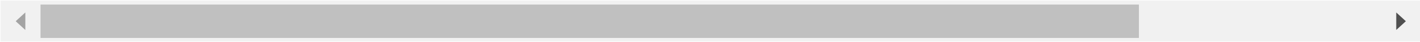

No urge to eat | Extremely strong urge to eat
1 | 100

How strong is your urge to eat this?

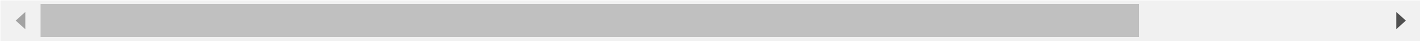

0566

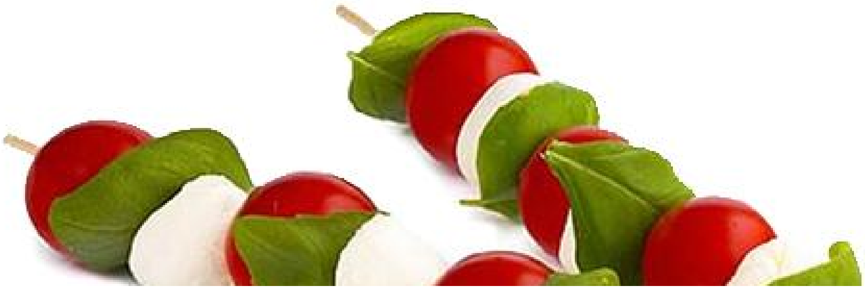

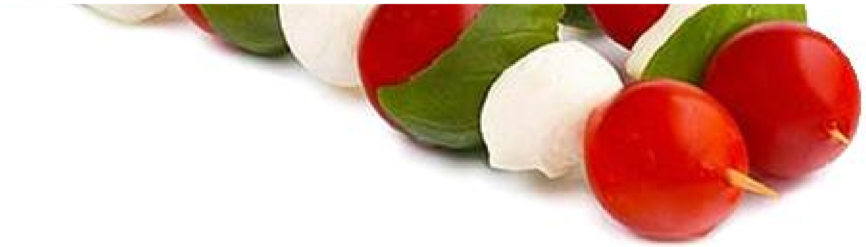

	Very Unpleasant	Very Pleasant
	1	100
How pleasant would it be to eat this?		

	No urge to eat	Extremely strong urge to eat
	1	100
How strong is your urge to eat this?		

Powered by Qualtrics
