# Supplementary material for: Transcranial direct current stimulation (tDCS) to dorsolateral prefrontal cortex influences perceived pleasantness of food
Source: Heliyon. 2023 Feb 4;9(2):e13275. doi: 10.1016/j.heliyon.2023.e13275 (PMC9929296; doi:10.1016/j.heliyon.2023.e13275)
Supplement: Multimedia component 2 [file mmc2.docx]

# Supplemental online materials

This online supplemental materials section includes additional information for Anderson et al manuscript.

In addition to the measures reported in the main manuscript, the following measures were collected: Measures of Motives Questionnaire (FCQ; Steptoe, Pollard, Wardle (1995), questions related to ethics and eating (Lindeman & Vaananen; 2000), and a social behavior questionnaire were also complete. Eye tracking data was also collected but not analyzed here.

## Food picture stimuli

During the experiment, participants viewed 40 food pictures from the ‘Food-pict’ database (Blechert et al., 2014). The pictures used in the present experiment are listed below.

| Food-pict image number | Item description |
| --- | --- |
| 4 | chocolate cookie |
| 5 | cookie mix |
| 9 | waffles |
| 16 | pancakes |
| 25 | ice cream with chocolate beans |
| 33 | spaghetti with tomato sauce |
| 36 | some different cake wedges |
| 46 | french fries |
| 48 | chocolate muffin |
| 50 | donuts and pastries |
| 54 | cheese platter |
| 56 | chocolate and vanilla ice cream |
| 74 | chocolate cake |
| 82 | cheese platter |
| 89 | strawberry cake |
| 106 | Sacher cake |
| 107 | chocolate cake |
| 111 | bar of chocolate with nuts |
| 112 | opened bar of chocolate with nuts |
| 113 | chips |
| 114 | croissants |
| 115 | sundae (with raspberries) |
| 126 | pastries and donuts |
| 131 | pizza (veggie/cheese) |
| 141 | lasagna |
| 173 | chocolate bar filled with milk cream |
| 206 | raspberries |
| 209 | wildberries mix |
| 211 | strawberries |
| 217 | fruit mix: kiwi, cherries, strawberries… |
| 220 | fruit plate: melon, pineapple, grapes… |
| 291 | chocolate truffle |
| 304 | potato gratin |
| 325 | fruit salad |
| 363 | chips / French fries |
| 394 | grape, whites |
| 398 | cherries |
| 416 | pistachios |
| 472 | Cappuccino |
| 566 | tomato and mozzarella skewer |

## Ratings during baseline and tDCS phase

In the manuscript, we presented participants’ change in cravings from baseline to tDCS phase (Figure 1). Below in Figure SOM 1, raw scores are shown during baseline and tDCS phase for each participant (lines) and group means (bars).

Figure SOM 1. Participant ratings by condition


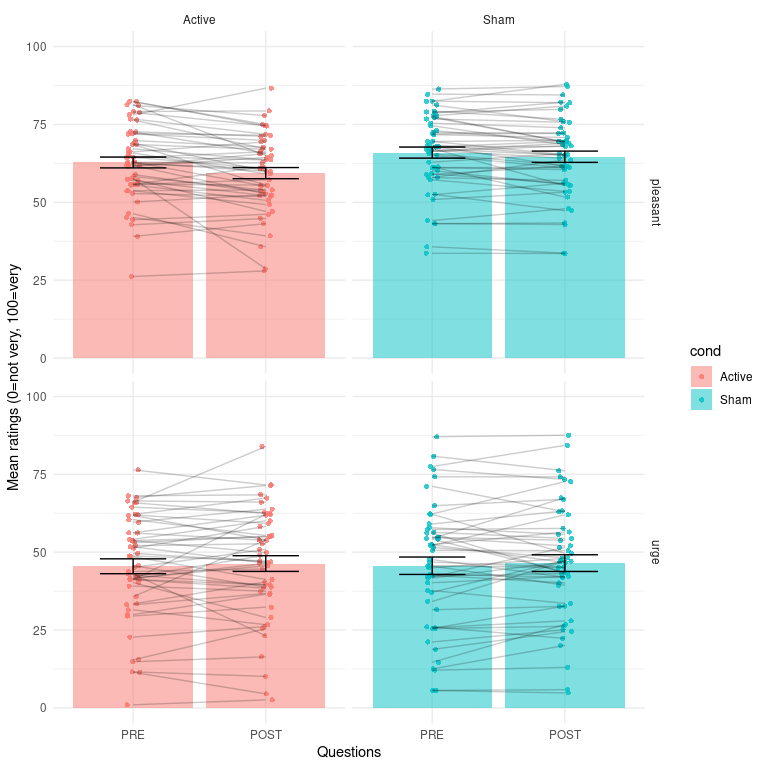


Note: Bars represent mean. Error bars represent standard errors. Each point represents an individual participant. Gray lines link participants’ baseline and stimulation phase ratings to show change in ratings. Pleasantness ratings were made on a scale where 1= very unpleasant; 100 = Very pleasant). Urge to eat ratings were made on a scale where 1 = No urge to eat; 100 = Extremely strong urge to eat.

## Subjective Hunger and Ratings

As described above, we explored how hunger might relate to the effect of tDCS. This analysis was exploratory, but we reasoned that hungry participants might be more likely to be influenced by tDCS because they have stronger cravings for food.

First, as one would expect, there was a strong relationship between self-reported hunger and both pleasantness (r=.32, 95% CI [.11, .50], t(82)=3.01, p=.003) and urge to eat (r=.48, 95% CI [.30, .63], t(82)=4.94, p<.001) at baseline, before any tDCS stimulation. Hungry participants had higher ratings of pleasantness and urge to eat (Figure SOM 2).

Figure SOM 2. Relationship between hunger and baseline picture ratings


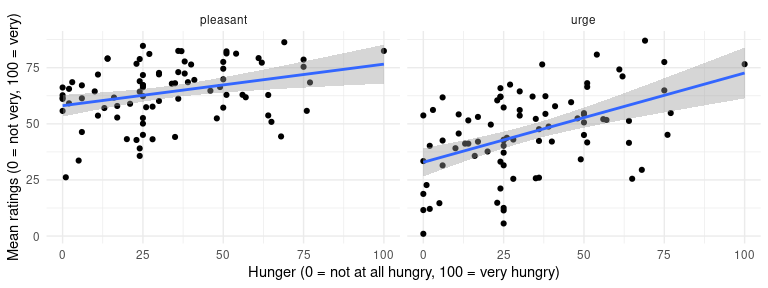


Note: Line represents linear model fitting the data. Ribbon represents 95% CI of linear model. Hunger ratings made on a scale where 0 = not at all hungry, 100 = very hungry. Pleasantness ratings were made on a scale where 1= Very unpleasant; 100 = Very pleasant). Urge to eat ratings were made on a scale where 1 = No urge to eat; 100 = Extremely strong urge to eat.

Next, we modeled the relationship between hunger and the effect of tDCS on food ratings. As before, change scores were calculated as ratings during the stimulation phase minus ratings during baseline phase. Linear models were used with changes in ratings as the dependent variable and hunger, tDCS condition (active vs. sham), and the interaction between hunger and tDCS condition were entered as predictors. In this analysis, neither the main effects or interaction were significant for ratings of pleasantness (all ps > .24) or urge to eat (all ps > .34). In short, the effect of tDCS did not depend on the level of hunger (see Figure SOM 3 for scatter plot and Table SOM1 for model summary).

Figure SOM 3. Effect of tDCS by hunger


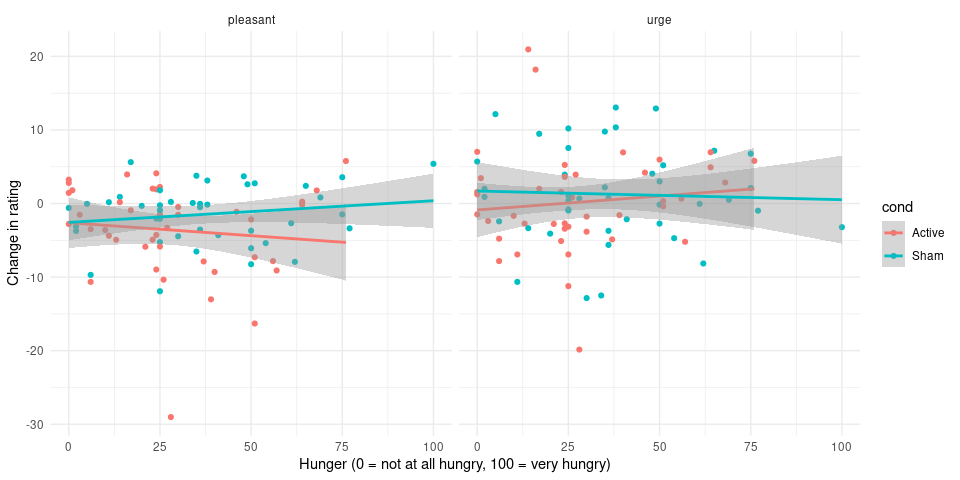


Note: Change in rating calculated as rating during the stimulation phase minus ratings during the baseline phase. Line represents linear model fitting the data. Ribbon represents 95% CI of linear model. Hunger ratings made on a scale where 0 = not at all hungry, 100 = very hungry. Pleasantness ratings were made on a scale where 1= Very unpleasant; 100 = Very pleasant). Urge to eat ratings were made on a scale where 1 = No urge to eat; 100 = Extremely strong urge to eat.

Table SOM 1: Output from hunger linear model

| **Characteristic** | **Beta** | **95% CI** | **p-value** |
| --- | --- | --- | --- |
| HR1_1 | -0.04 | -0.12, 0.05 | 0.4 |
| cond |  |  |  |
| Active |  |  |  |
| Sham | 0.04 | -4.2, 4.3 | >0.9 |
| HR1_1 * cond |  |  |  |
| HR1_1 * Sham | 0.06 | -0.04, 0.17 | 0.2 |

## Restrained Eating Questionnaire

The next section describes an exploratory analysis of the three factor eating questionnaire (Stunkard and Messick, 1985) and changes in ratings during tDCS. The three factors are described as: 1) cognitive control over eating (e.g., “I consciously hold back at meals in order not to gain weight”); 2) controlling urges (e.g., “Sometimes things just taste so good that I keep on eating even when I am no longer hungry”); and 3) susceptibility to hunger (e.g., “Dieting is hard for me because I just get too hungry”). Figures SOM 4 - 6 visually show the relationships and Tables SOM 2 - 4 present the linear models below.

Figure SOM 4: Effect of tDCS by Factor 1 (cognitive control of eating behavior)


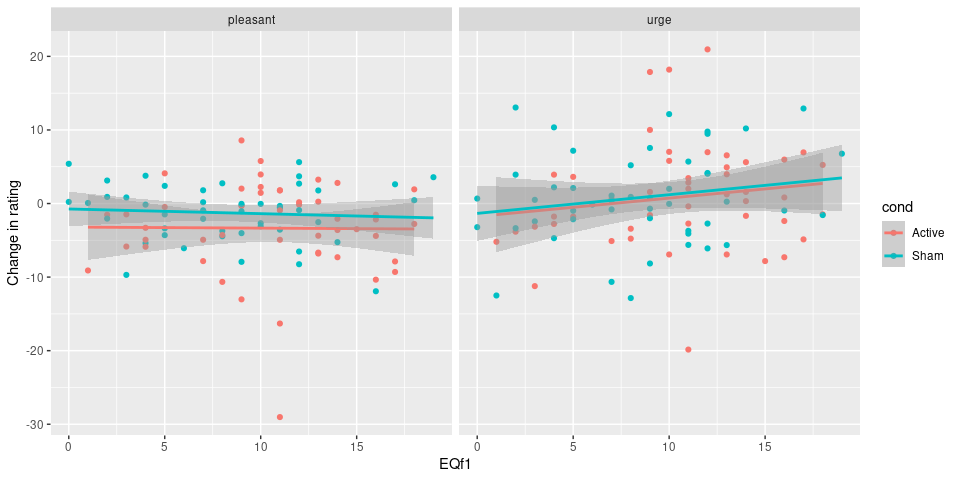


Figure SOM 5: Effect of tDCS by Factor 2 (disinhibition of control)


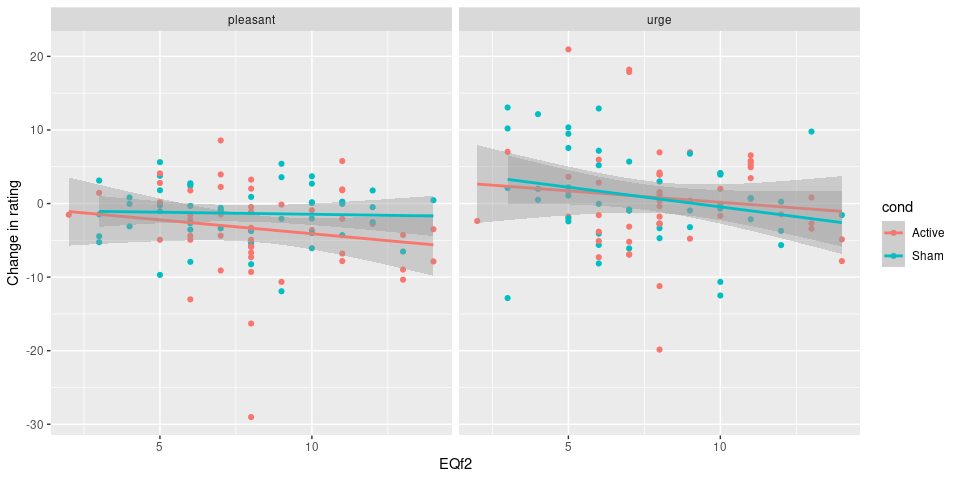


Figure SOM 6: Effect of tDCS by Factor 3 (susceptibility to hunger)


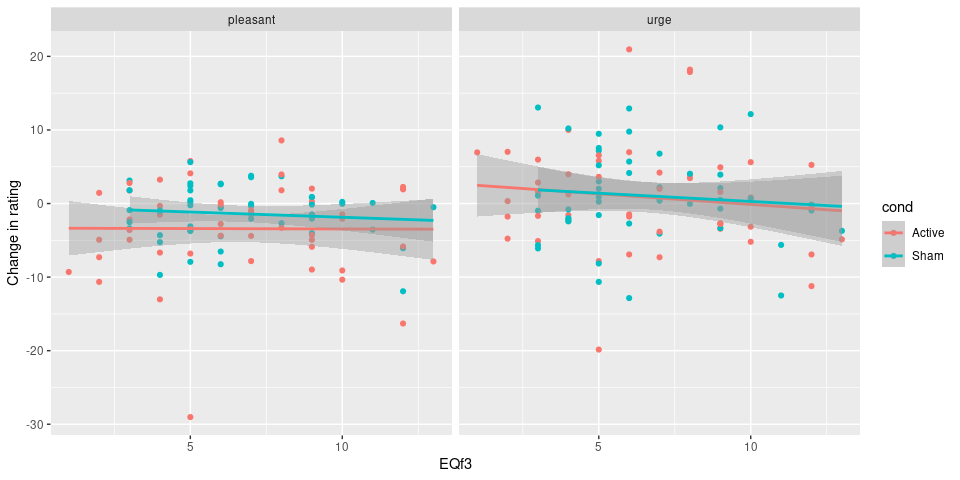


To explore whether these factors moderated the effects of tDCS, linear models were used with changes in ratings as the dependent variable and restrained eating, tDCS condition (active vs. sham), and the interaction between restrained and tDCS condition as the predictors. We ran one model for each factor of restrained eating (cognitive control over eating; controlling urges; susceptibility to hunger. In this analysis neither the main effects or interactions were significant for ratings of pleasantness or urge to eat for any of the three factors (see tests below). In short, the effect of tDCS did not depend on scores on the three factor of restrained eating.

Table SOM 2 - 7: Linear models predicting liking and wanting from restrained eating

LM predicting pleasantness ratings by condition and factor 1 (cognitive control over eating)

| **Characteristic** | **Beta** | **95% CI** | **p-value** |
| --- | --- | --- | --- |
| EQf1 | -0.01 | -0.36, 0.33 | >0.9 |
| cond |  |  |  |
| Active |  |  |  |
| Sham | 2.5 | -2.6, 7.5 | 0.3 |
| EQf1 * cond |  |  |  |
| EQf1 * Sham | -0.05 | -0.52, 0.43 | 0.8 |
|  |  |  |  |
|  |  |  |  |

LM predicting pleasantness ratings by condition and factor 2 (controlling urges)

| **Characteristic** | **Beta** | **95% CI** | **p-value** |
| --- | --- | --- | --- |
| EQf2 | -0.38 | -0.92, 0.17 | 0.2 |
| cond |  |  |  |
| Active |  |  |  |
| Sham | -0.57 | -6.9, 5.8 | 0.9 |
| EQf2 * cond |  |  |  |
| EQf2 * Sham | 0.32 | -0.43, 1.1 | 0.4 |
|  |  |  |  |
|  |  |  |  |

LM predicting pleasantness ratings by condition and factor 3 (susceptibility to hunger)

| **Characteristic** | **Beta** | **95% CI** | **p-value** |
| --- | --- | --- | --- |
| EQf3 | -0.01 | -0.49, 0.47 | >0.9 |
| cond |  |  |  |
| Active |  |  |  |
| Sham | 2.9 | -2.4, 8.2 | 0.3 |
| EQf3 * cond |  |  |  |
| EQf3 * Sham | -0.13 | -0.88, 0.61 | 0.7 |
|  |  |  |  |

Urge
LM predicting urge to eat ratings by condition and factor 1 (cognitive control over eating)

| **Characteristic** | **Beta** | **95% CI** | **p-value** |
| --- | --- | --- | --- |
| EQf1 | 0.25 | -0.19, 0.69 | 0.3 |
| cond |  |  |  |
| Active |  |  |  |
| Sham | 0.42 | -6.1, 6.9 | 0.9 |
| EQf1 * cond |  |  |  |
| EQf1 * Sham | 0.00 | -0.61, 0.61 | >0.9 |
|  |  |  |  |

LM predicting urge to eat ratings by condition and factor 2 (controlling urges)

| **Characteristic** | **Beta** | **95% CI** | **p-value** |
| --- | --- | --- | --- |
| EQf2 | -0.31 | -1.0, 0.39 | 0.4 |
| cond |  |  |  |
| Active |  |  |  |
| Sham | 1.6 | -6.5, 9.7 | 0.7 |
| EQf2 * cond |  |  |  |
| EQf2 * Sham | -0.22 | -1.2, 0.73 | 0.6 |
|  |  |  |  |

LM predicting urge to eat ratings by condition and factor 3 (susceptibility to hunger)

| **Characteristic** | **Beta** | **95% CI** | **p-value** |
| --- | --- | --- | --- |
| EQf3 | -0.29 | -0.91, 0.33 | 0.4 |
| cond |  |  |  |
| Active |  |  |  |
| Sham | -0.24 | -7.2, 6.7 | >0.9 |
| EQf3 * cond |  |  |  |
| EQf3 * Sham | 0.06 | -0.90, 1.0 | 0.9 |

##

## Trait and State Food Craving

The next section describes an exploratory analysis of the two factor Food Cravings questionnaire (Nijs et al., 2007) and changes in ratings during tDCS. The two factors measured by this scale are are trait cravings and state cravings. Figures SOM 7 and 8 visually show the relationships and linear models are tested below.

Figure SOM 7: Effect of tDCS by Trait Food Craving


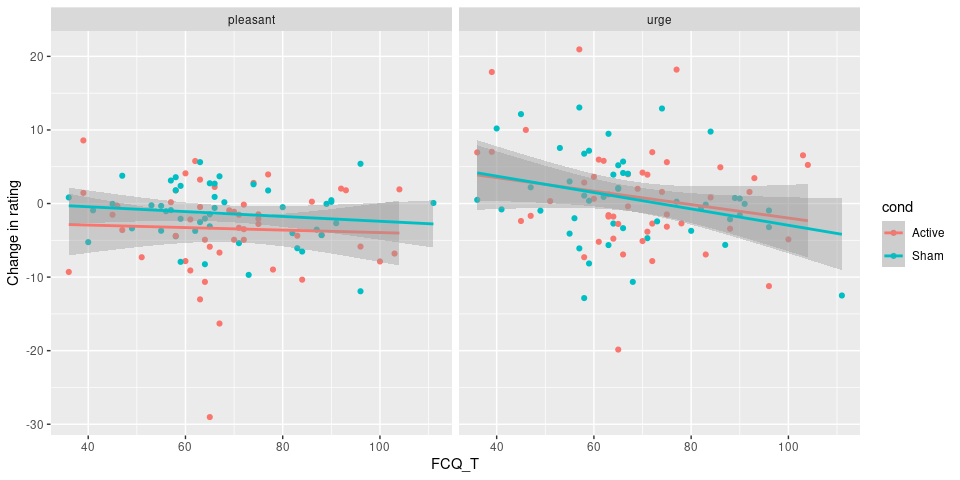


Figure SOM 8: Effect of tDCS by State Food Craving


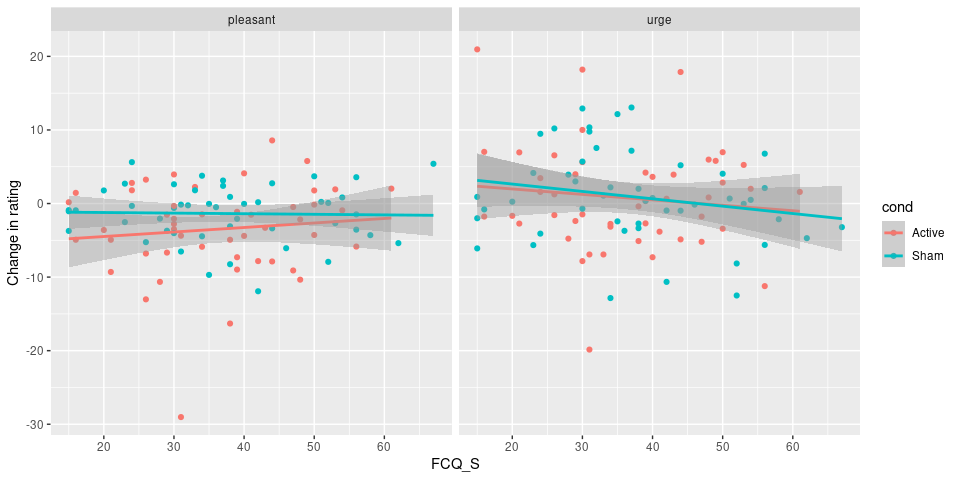


To explore whether these factors moderated the effects of tDCS, linear models were used with changes in ratings as the dependent variable and craving, tDCS condition (active vs. sham), and the interaction between craving and tDCS condition as the predictors. We ran one model for each factor of craving (trait and state). In this analysis neither the main effects or interactions were significant for ratings of pleasantness or urge to eat for either factor (see tests below). In short, the effect of tDCS did not depend on scores on the craving factors.

Table SOM 8 - 11: Linear models predicting liking and wanting from trait and state cravings

LM predicting pleasantness ratings by condition and trait cravings

| **Characteristic** | **Beta** | **95% CI** | **p-value** |
| --- | --- | --- | --- |
| FCQ_T | -0.02 | -0.11, 0.08 | 0.7 |
| cond |  |  |  |
| Active |  |  |  |
| Sham | 3.1 | -6.2, 12 | 0.5 |
| FCQ_T * cond |  |  |  |
| FCQ_T * Sham | -0.02 | -0.15, 0.12 | 0.8 |
|  |  |  |  |
|  |  |  |  |

LM predicting urge to eat ratings by condition and trait cravings

| **Characteristic** | **Beta** | **95% CI** | **p-value** |
| --- | --- | --- | --- |
| FCQ_T | -0.09 | -0.21, 0.03 | 0.12 |
| cond |  |  |  |
| Active |  |  |  |
| Sham | 1.0 | -11, 13 | 0.9 |
| FCQ_T * cond |  |  |  |
| FCQ_T * Sham | -0.02 | -0.18, 0.14 | 0.8 |
|  |  |  |  |

LM predicting pleasantness ratings by condition and state cravings

| **Characteristic** | **Beta** | **95% CI** | **p-value** |
| --- | --- | --- | --- |
| FCQ_S | 0.06 | -0.07, 0.19 | 0.4 |
| cond |  |  |  |
| Active |  |  |  |
| Sham | 4.7 | -2.2, 11 | 0.2 |
| FCQ_S * cond |  |  |  |
| FCQ_S * Sham | -0.07 | -0.25, 0.11 | 0.4 |
|  |  |  |  |

LM predicting urge to eat ratings by condition and state cravings

| **Characteristic** | **Beta** | **95% CI** | **p-value** |
| --- | --- | --- | --- |
| FCQ_S | -0.07 | -0.24, 0.10 | 0.4 |
| cond |  |  |  |
| Active |  |  |  |
| Sham | 1.2 | -7.6, 10.0 | 0.8 |
| FCQ_S * cond |  |  |  |
| FCQ_S * Sham | -0.03 | -0.25, 0.20 | 0.8 |
